# Optimizing the implementation of a forest fuel break network

**DOI:** 10.1371/journal.pone.0295392

**Published:** 2023-12-13

**Authors:** Alan A. Ager, Michelle A. Day, Bruno A. Aparício, Rachel Houtman, Andrew Stinchfield

**Affiliations:** 1 USDA Forest Service, Rocky Mountain Research Station, Missoula Fire Sciences Lab, Missoula, Montana, United States of America; 2 International Visiting Scholar, USDA Forest Service, Rocky Mountain Research Station, Missoula Fire Sciences Lab, Missoula, Montana, United States of America; 3 Oregon State University, College of Forestry, Forest Ecosystems & Society, Corvallis, Oregon, United States of America; 4 USDA Forest Service, Umatilla National Forest, Pendleton, Oregon, United States of America; University of British Columbia, CANADA

## Abstract

Methods and models to design, prioritize and evaluate fuel break networks have potential application in many fire-prone ecosystems where major increases in fuel management investments are planned in response to growing incidence of wildfires. A key question facing managers is how to scale treatments into manageable project areas that meet operational and administrative constraints, and then prioritize their implementation over time to maximize fire management outcomes. We developed and tested a spatial modeling system to optimize the implementation of a proposed 3,538 km fuel break network and explore tradeoffs between two implementation strategies on a 0.5 million ha national forest in the western US. We segmented the network into 2,766 treatment units and used a spatial optimization model to compare linear versus radial project implementation geometries. We hypothesized that linear projects were more efficient at intercepting individual fire events over larger spatial domains, whereas radial projects conferred a higher level of network redundancy in terms of the length of the fuel break exposed to fires. We simulated implementation of the alternative project geometries and then examined fuel break-wildfire spatial interactions using a library of simulated fires developed in prior work. The results supported the hypothesis, with linear projects exhibiting substantially greater efficiency in terms of intercepting fires over larger areas, whereas radial projects had a higher interception length given a fire encountered a project. Adding economic objectives made it more difficult to obtain alternative project geometries, but substantially increased net revenue from harvested trees. We discuss how the model and results can be used to further understand decision tradeoffs and optimize the implementation of planned fuel break networks in conjunction with landscape conservation, protection, and restoration management in fire prone regions.

## Introduction

Methods and models to design, prioritize and evaluate fuel break networks have potential global application where major increases in fuel management investments are planned in response to a growing incidence of wildfires. Proposals to build new and expand existing linear fuel breaks networks (FBN) have emerged as part of several national initiatives in the US and elsewhere [1–4] in response to wildland fire events that are increasingly challenging the efficiency of suppression operations and causing tragic loss of human life [5–9]. Here, we distinguish FBNs as a subset of many alternative spatial treatment strategies where segments of reduced fuel loadings are interconnected to create a network, and are typically built on a core of existing roads to minimize cost and maximize fire control, ingress, egress and safety [10,11]. Natural barriers are also used, including low-flammability vegetation [12], and non-vegetated features such as lakes and rivers. Fire suppression modeling using historic wildfire events can also be used to locate FBN segments [13]. Mechanical treatments to reduce fuels where necessary within FBNs include thinning, mastication, mowing, grinding, and discing [3]. Treatments also include re-vegetating plowed areas with fire resistant vegetation to create green fuel breaks where the composition and moisture in the fuels substantially slows fire spread [3]. It is widely recognized that fuel breaks are unlikely to halt fire progression without suppression resources [14,15], and on average their effectiveness is around 60% as measured in the sagebrush biome [16]. The availability of suppression resources are highly variable and dependent on local fire behavior at the point of encounter with the fuel break [17].

Application of FBN strategies spans diverse fire regimes and underlying fuel types ranging from grass, shrub steppe, Mediterranean shrub, pine plantations, and boreal forests [4,10,18–22]. For instance, US federal land management agencies are implementing 17,700 km of fuel breaks on 90 million ha in the western US to manage the invasive grass-fire cycle in sagebrush steppe, where fire is increasingly threatening habitat for the endangered sage grouse [3]. In China, FBNs have been used in the boreal forests since 1988 in the Heilongjiang Province to help reduce negative impacts from fires [4]. In Portugal, a new 10-year fuel management plan [1] calls for the implementation of a nation-wide FBN covering 3,538 km that traverses multiple fire regimes, peri-urban landscapes interspersed with forest plantations, and abandoned agricultural areas. Similar efforts and strategies to containerize landscapes with FBNs exist in all European Union fire-prone regions [19,22,23]. It is safe to assume that expansion of existing and building of new fuel break networks as a fire protection strategy, in concert with other landscape treatment strategies, will only accelerate in the future as climate change and urban encroachment into wildlands exacerbate human risk from wildland fire.

Mapping FBNs is largely left to local expert opinion and considers historic fire regime, predominant wind direction, fuel type, proximity to communities, local ecological impacts, and cost [10,19,22]. More recently, mapping the optimal locations for fuel breaks was also assessed using predicted patterns of fire behavior, access to suppression resources, containment probability and other factors [13]. However, localized methods for delineating FBNs that span hundreds of kilometers over millions of hectares based on expert opinion could be improved with optimization models that are able to analyze design alternatives (density, width, treatment methods) [4,23] and predict effectiveness under stochastic wildfire futures [24]. Important tradeoffs among design parameters are not well studied, and thus generalities are yet to be arrived at due to the diverse fire systems in which FBNs are employed. For instance, given a fixed budget and specifications for residual fuels [22], site-specific treatments can be allocated and optimized based on expected fire behavior, with wider firebreaks being used in forests (e.g., coniferous forests) where there is higher potential for crown fires, and narrower firebreaks used to reduce fire spread in a fuels that typically propagate only surface fires [22,25]. At larger scales, optimizing fuel break networks requires some understanding of how changing network density (km/km^2^) versus the width of the individual fuel break segments affects network cost and effectiveness. In one recent simulation study in boreal forests it was concluded that for a given total treated area, higher density networks at narrow widths were more effective at reducing burn probability than lower density, wider networks widths [4], consistent with prior findings [21,23].

Given that major initiatives are being formulated to build new and expand existing FBNs, models and tools are needed to both design and map optimal networks, and plan their implementation over space and time. Proposed national or regional FBNs will take many years to implement on lands that have inherently wide variation in cost effectiveness and ecological impacts [26,27]. Despite many studies to optimally locate fuel breaks dispersed across landscapes [28–30], there are few case studies where proposed FBNs have undergone rigorous evaluation in terms of tradeoffs, feasibility, and optimal prioritization and implementation strategies [4,24]. The importance of spatial prioritization in restoration, risk reduction, and conservation in terms of achieving outcomes has been widely discussed in prior literature [31–33]. Fuel break networks on national forests in the western US will be constructed with a series of sequential projects over the next 10–20 years to broadly meet logistical, administrative, and planning guidelines, with each project treating hundreds to thousands of hectares to connect fuel break segments. The analytical problem at hand is to create a strategic plan with defined priorities and treatment schedules that can be used to estimate required funding, workforce, machinery, as well as other inputs and outputs including net revenue from harvesting operations.

To address selected gaps described above, we studied the effect of alternative prioritization scenarios for a proposed FBN on a western US forested landscape that, like many areas globally, has experienced a significant increase in large, severe wildfire in the last three decades. The study area was the Umatilla National Forest, where staff mapped a 3,538 km fuel break network using expert opinion from local fire management experts. The original purpose of the delineation was an exercise in fire planning to identify control locations in advance of fire events. However, assessment of the fuel conditions and potential fire behavior by UNF staff indicated the need for substantial forest and fuel management activities over 10–20 years to render the network safe for suppression activities. The UNF needs a longer-term prioritization plan to identify what segments should be built first, and how an overall implementation strategy can be optimized in terms of both economics and improving fire management.

We used a scenario planning model to analyze alternative implementation scenarios where treatment segments were organized into planning areas and scheduled using divergent geometries and management priorities. For instance, what are the tradeoffs between implementing treatments in a radial geometry, i.e. a dense, semi-circular web of treatments that radiate from the project centroid, or alternatively as linear segments that span longer distances, and how does geometry affect the attainment of other forest management objectives, including generating revenue for larger landscape restoration projects. These design alternatives are not analyzed in conventional project planning and prioritization since landscape (versus fuel break) treatments are generally dispersed throughout project areas to meet broad restoration and resiliency goals [34]. We discuss the results in terms of prioritizing proposed large scale networks in concert with extensive forest and fuel management focused on restoring resiliency in the fire excluded forests in the western US.

## Materials and methods

### Study area

**Fig 1:**
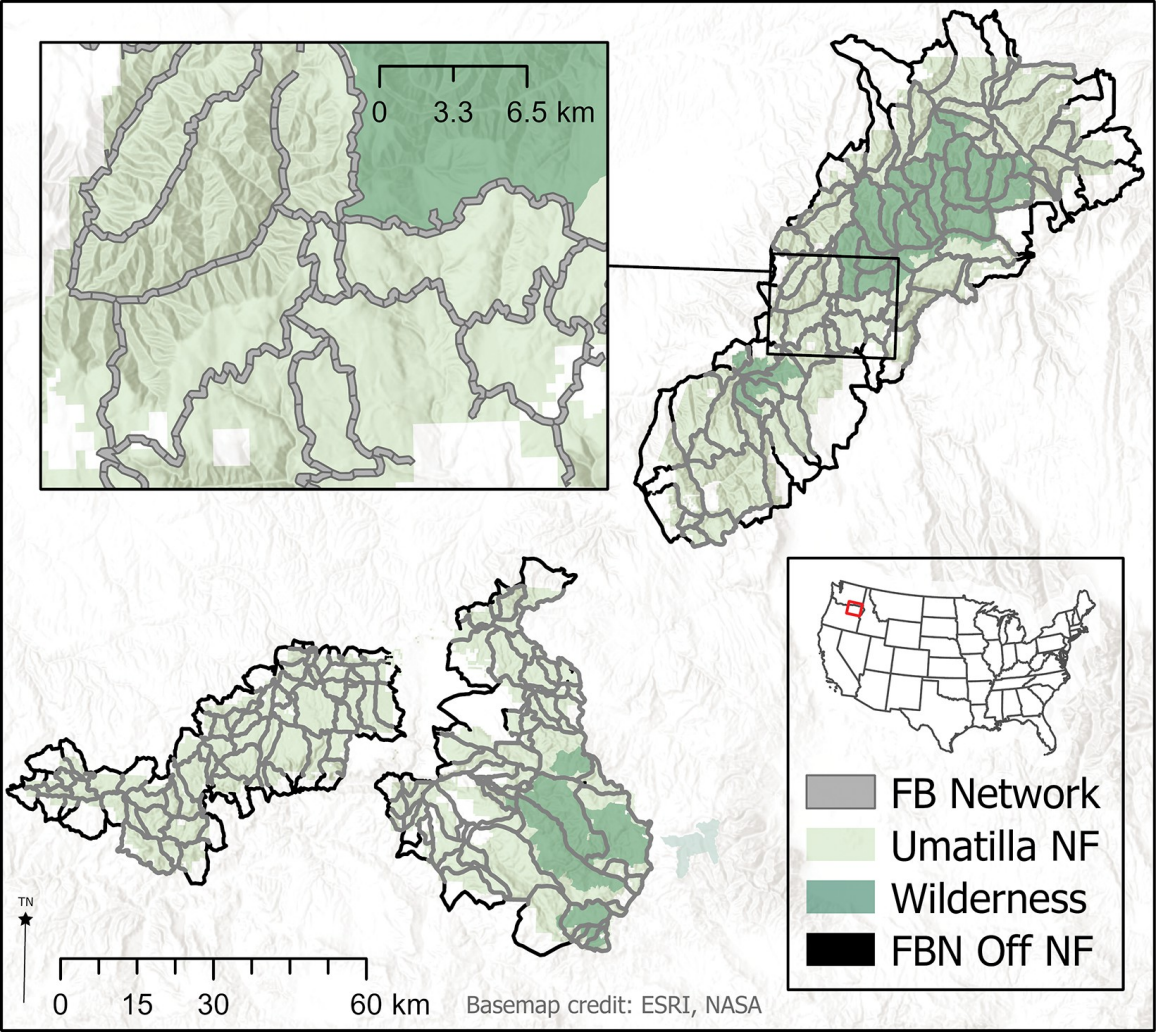
Study area showing the proposed fuel break network (FBN) and the Umatilla National Forest. Note that the FBN was designed by planners to extend beyond the boundaries of the national forest and within federally designated wilderness areas. The current study analyzed the portion of the FBN on the Umatilla National Forest and available for management under the current forest plan. Black fuel break network segments (FBN Off NF) are on private land.

The study area was the 520,000 ha Umatilla National Forest (henceforth UNF) located in the Blue Mountain ecoregion [35] within northeast Oregon and southeast Washington State (Fig 1). Elevations generally range from 900 m to 1,500 m, with higher peaks close to 3,000 m. Dry forests of ponderosa pine (*Pinus ponderosa* Lawson & C. Lawson) dominate lower elevations, with dry mixed conifer (grand fir (*Abies grandis* (Douglas ex D. Don) Lindl) and Douglas-fir (*Pseudotsuga menziesii* (Mirb.) Franco)) at higher elevations. Cold dry forested areas are dominated by lodgepole pine (*Pinus contorta* Douglas ex Loudon) at higher elevations. Basalt scablands and extensive grasslands are common along west facing steep canyon lands where soil moisture limits the development of vegetation [36].

Wildfires on the national forest are common with an average of 94 ignitions per year, with a mean fire size of 52 ha, calculated over the period 1992–2020 [37]. The largest fire burning on the UNF during this period was the Columbia Complex (44,215 ha). Other significant fires over the last 28 years include Butte Creek (32,460 ha), Red Hill (21,630 ha), School (21,043 ha), Monument Complex (13,092 ha), and Green Ridge (17,188 ha).

### Fuel break network

The UNF designed a fuel break network in 2020 using expert opinion from local fire management staff. The FBN consisted of 1,752 km within the UNF boundary and 1,787 km on adjacent lands, with a planned 150 m buffer from the road centerline (300 m width total) to allow for suppression and other fire management activities. The 300 m FBN width was established by federal legislation [38] and is consistent with recommendations in prior literature [39–41].

**Fig 2:**
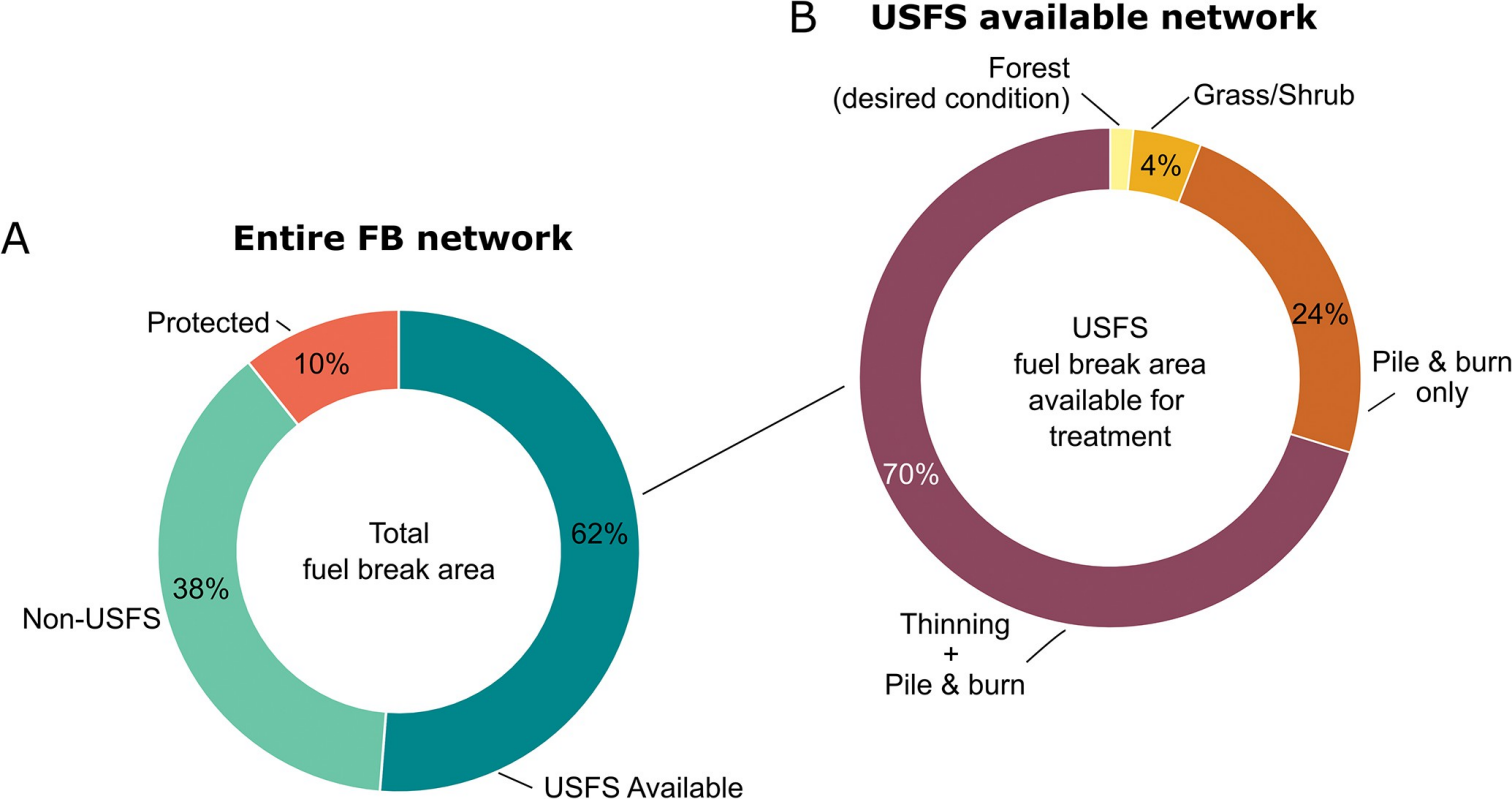
Ownership and treatment composition of the proposed fuel break network. A) Proportion of the fuel break network area by ownership and protection status. B) Proportion requiring thinning and pile burn treatments for segments that are on the Umatilla National Forest (UNF) and not in protected areas (USFS Available), as determined from processing forest inventory data with the Forest Vegetation Simulator. See Methods section for details on fuel treatment prescriptions. Note data in (A) represent the entire network as originally mapped both on and off the UNF, whereas the simulated treatments and response targeted the subset of land on the UNF and outside of protected areas as shown in (B). The total area in (A) is 109,659 ha, or 3,538 km linear distance of fuel treatments. Associated data are in Table S2.1 in S2 Appendix.

The total area within the buffered network inside and outside the UNF, including roads used to delineate the network, was 109,659 ha with 67,496 ha on the UNF (62%). Within the UNF, 56,198 ha (83%) were available for management on the UNF, meaning they were outside of protected areas (Fig 2A). Fuel breaks wholly contained within non-Forest Service or protected areas were not analyzed in the study, although we note that these segments will add considerable complexity to the implementation since mechanical treatments are prohibited in protected areas, and implementing treatments on non-federal lands are not guaranteed. The area of the roadbed was included in the estimate since they are delineated as a line feature, and thus the area of vegetation was overestimated by about 3%. About 2% of the entire FBN area was classified as non-conifer forest (Fig 2A) and was not considered for treatments. Note that non-conifer forests are generally basalt scablands and grasslands that do not support the development of significant fuel loadings.

### Modeling fuel treatments in fuel break segments

**Fig 3:**
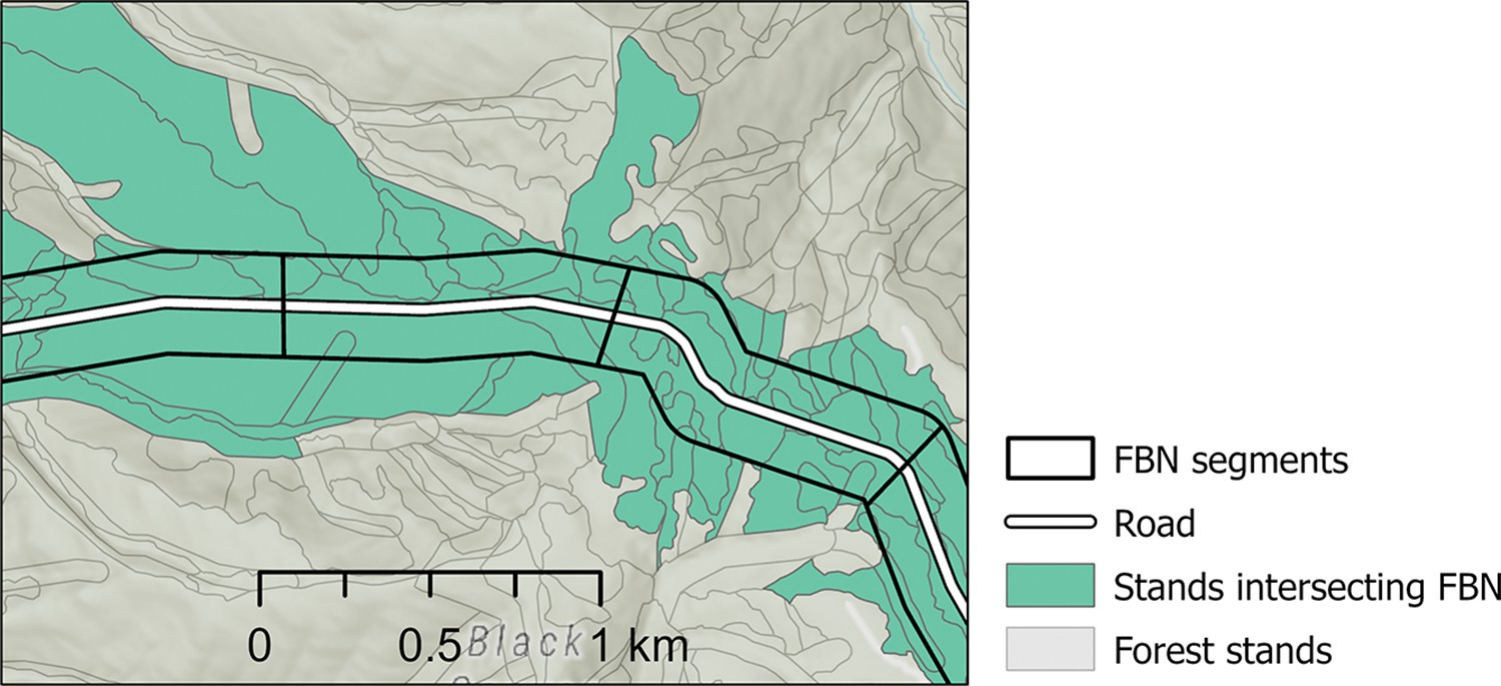
Example fuel break network (FBN) section. Umatilla National Forest stand polygons delineated as part of the forest vegetation inventory data were assigned to each segment.

We created 2,766 treatment units by subdividing the FBN into 1,000-m long x 300-m wide segments. Segments were intersected with the UNF’s vegetation polygon layer (n = 25,137 polygons intersecting the segments) and attributed with the associated tree inventory data maintained in the UNF’s FSVeg database (Fig 3) (S1 Appendix) [42]. The inventory data included trees per ha by species and diameter measured at breast height (1.6 m above ground). The inventory consisted of measured field plot data collected using the Forest Service FSVeg protocol [42]. Polygons lacking current stand exam data were assigned inventory data using statistical imputation [43] automated within the FSVeg system.

Potential treatments for each segment were simulated with the Forest Vegetation Simulator (FVS) Blue Mountains variant [44] and consisted of thinning all stands that exceeded 15% forest canopy cover. The thinning prescription was developed by UNF staff specifically for the FBN with the objective of reducing crown fuels significantly below thresholds for crown fire behavior [45]. Simulated thinning prioritized the removal of smaller trees of fire-intolerant species (e.g., grand fir) to reduce ladder fuels that contribute to torching and crowning fire behavior. The maximum tree size for harvest was set at 53.3 cm diameter at breast height for all species excluding grand fir to conform to harvest guidelines on eastern Oregon national forests [46]. The maximum tree size for harvest of grand fir was set at 76.2 cm, as specified under the amended UNF plan guidelines [47]. Thin-from-below treatments were followed with simulated slash disposal by using the FVS FUELMOVE keyword [48], which has the same effect as the pile burn process in terms of removing fuels from the site. This treatment assumed both hand and machine piling of harvest residue and downed woody material. We also identified stands requiring surface fuel treatment without thinning as those with less than 15% canopy closure and predicted fire behavior estimated from FSim [49] wildfire simulation outputs [50]. Specifically, if greater than 20% of simulated fires that encountered the fuel break segment exhibited a flame length > 1.2 m the segment was identified for surface fuel treatment (Fig S1.1 in S1 Appendix). FVS outputs included estimated merchantable thin volume per cubic feet by species and diameter for each FBN segment. Volume was converted to metric (m^3^) and used to calculate net revenue as described below. Note that the prescriptions used in this study for the FBN differ significantly from those used in local forest restoration projects where thinning is based on stand density index thresholds [51] and broadcast burning is used in the dry forest vegetation group [26].

### Modeling alternative project geometries

**Fig 4:**
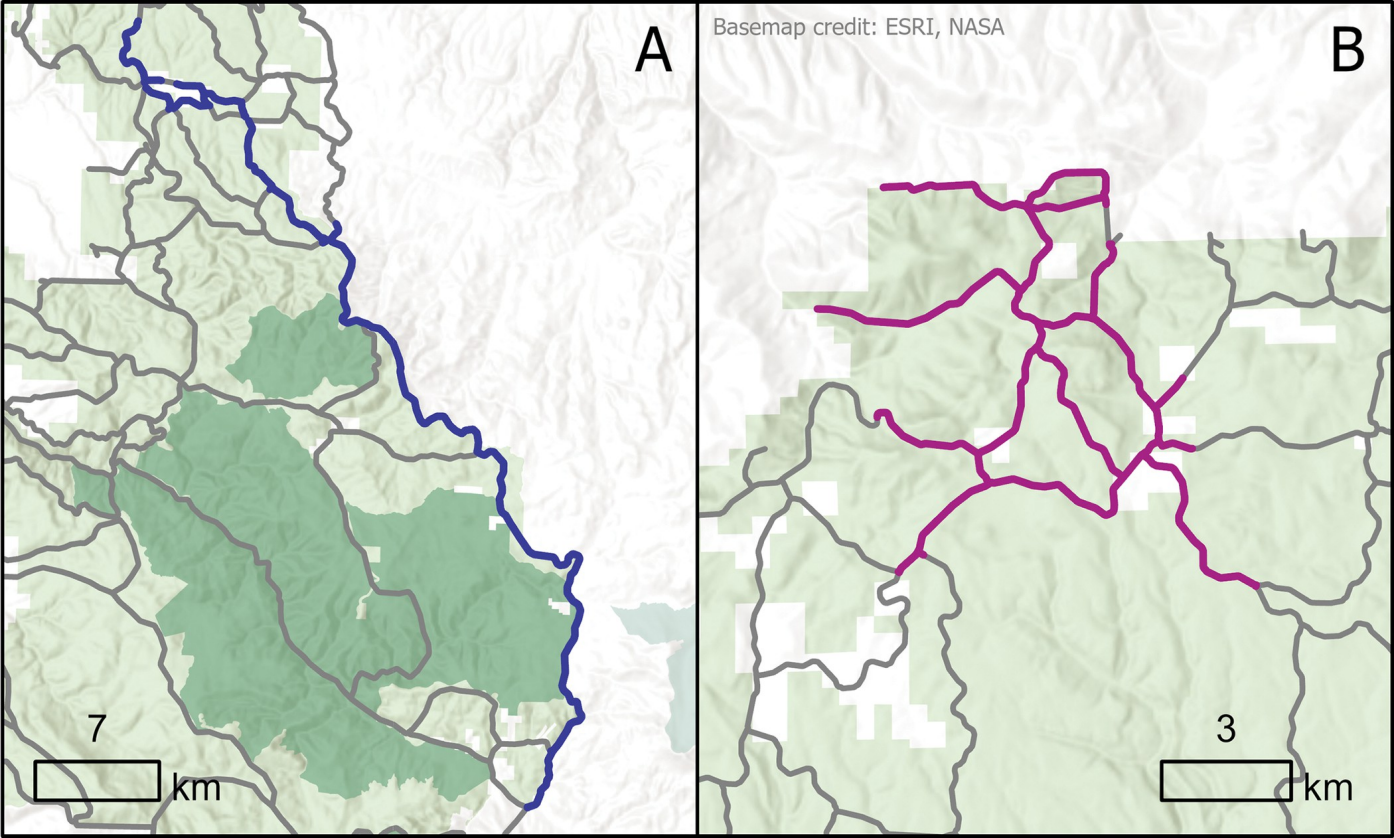
Example project geometries analyzed in the study. Linear fuel break network (FBN) project (A) has a sinuosity value of 1.5 and a linear distance of 73 km. Radial FBN project (B) has a sinuosity value of 4.36 and a linear distance of 50 km. Each project treated approximately the same area. We hypothesized that radial projects have a higher overlap with a given fire, whereas linear projects have a higher probability of encounter with a random wildfire over a larger spatial domain on the Umatilla National Forest. See Fig 5 for a description of the sinuosity index.

As described above, the UNF plans to implement the FBN over the next 10–20 years in a sequence of prioritized project areas, each treating between 800–1,200 ha. There are two divergent spatial strategies being considered to implement individual fuel break projects: 1) fragment the forests into increasingly smaller parcels by implementing projects that consist of long linear fuel breaks (Fig 4A); or 2) implement projects by treating all interconnected fuel breaks thus creating high density radial networks one project at a time (Fig 4B). To analyze the cost and benefits of these alternative scenarios we first created sequences of radial versus linear projects using the ForSys planning model (S1 Appendix) [26,52]. We set the treatment goal between 800 and 1,200 ha per project, or approximately 30 km of fuel breaks. Linear projects were created by maximizing the distance between adjoining segments within each project and radial projects were created by aggregating adjacent network segments based on distance to the project centroid. The model used every segment as a seed, building projects by adding segments to maximize the distance objective until the project area reached a maximum of 1,200 ha. This process was repeated using the remaining segments that were not assigned to projects in previous interactions until the remaining segments could not meet the minimum area constraint. Initial testing showed that as optimal projects were built it became increasingly difficult for the algorithm to aggregate segments to meet the 1,200-ha target due to the dispersion of available segments and the irregular UNF boundary.

To understand how optimizing alternative geometries might affect the net revenues from harvesting trees and treating fuels we simulated two additional scenarios that used an objective function weighted with the sum of revenue and distance for a total of four scenarios (Table 1). For this purpose, we standardized the revenue and distance by converting to percent of the maximum values. We note that in prior work with the ForSys model we explored a range of weighting schemes to quantify revenue tradeoffs [53] but excluded this line of inquiry from the current study largely for space reasons.

**Table 1:** Description of scenarios and selection criteria to build project areas from fuel break segments.

Scenario objective for creating projects	Name	Scenario number	Selection criteria for adding adjacent segments to build a project area
Radial shape	Radial	1	Minimize distance to project centroid
Radial shape and maximize revenue	Radial + Revenue	2	Maximize revenue and minimize distance to project centroid
Linear shape	Linear	3	Maximize distance to project centroid
Linear shape and maximize revenue	Linear + Revenue	4	Maximize both revenue and distance to project centroid

### Response variables

**Fig 5:**
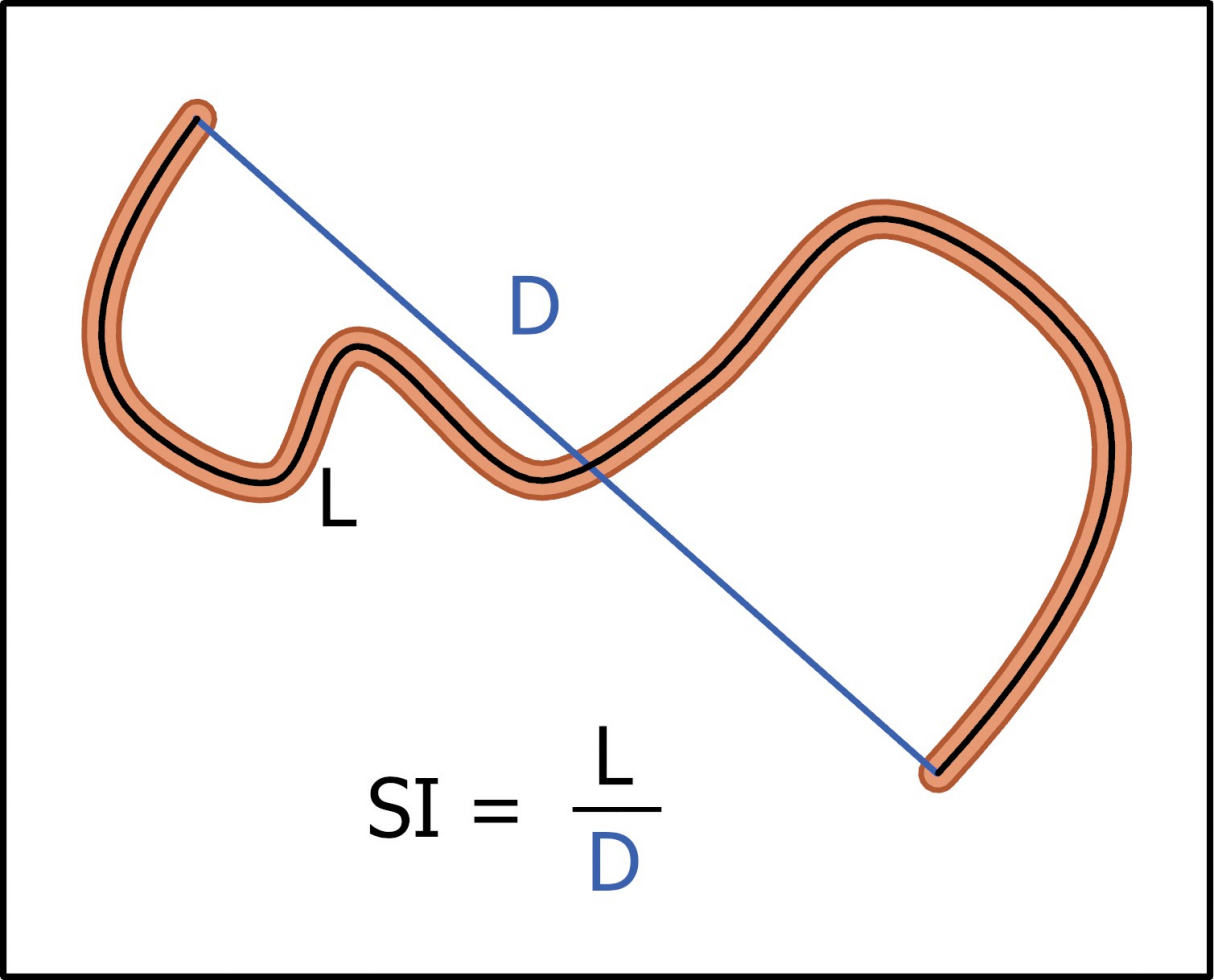
Sinuosity index (SI). SI, also called tortuosity [54], is the ratio of the total length along the network and the Euclidean distance between the endpoints. High values indicate a more sinuous geometry.

The linearity of the resulting projects was measured with a sinuosity index calculated as the ratio between the total length of the FBN project and the straight line that connects the two farthest points [54] (Fig 5). A high sinuosity index represents a radial shape network of treatments whereas low values are found for linear projects. A sinuosity value of 1 represents a straight line, which is likely impossible to obtain in montane landscapes.

**Fig 6:**
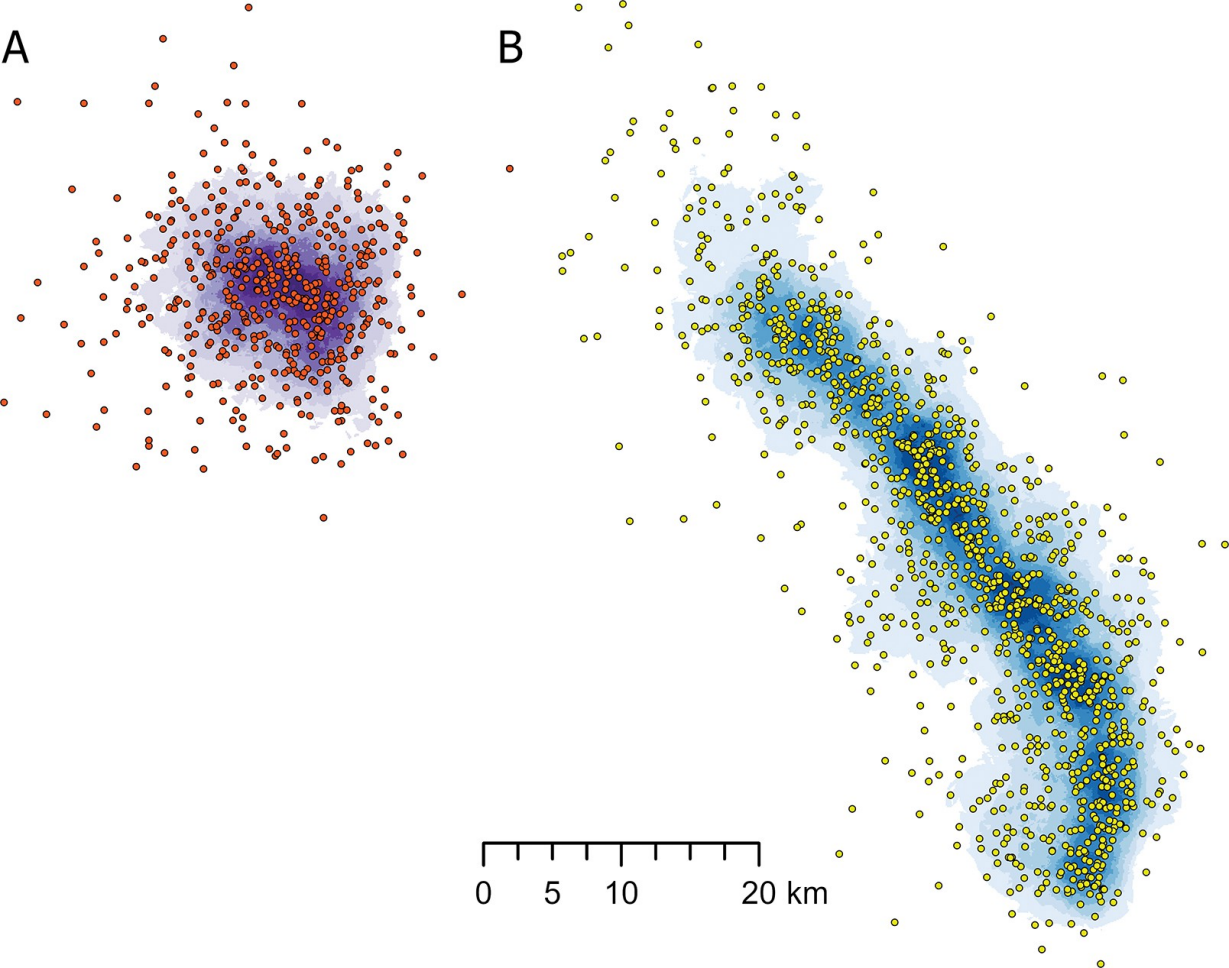
Example of two firesheds created by optimizing different geometries A) radial and B) linear. Firesheds were defined as the area that encloses 88% of the ignitions that encounter the fuel breaks within a simulated project area.

The efficiency of projects to intercept fires was measured using a library of simulated fires (n = 32,894) from the FSim fire model completed by the UNF as part of prior prioritization work [26,50,55]. We intersected all simulated fire perimeters with the FBN and calculated: 1) the total fires encountered per project, 2) the total length of intersection between the FBN and the fire perimeter per area burned in the project, 3) the total area burned by fires that encountered fuel breaks in each project, and 4) the area surrounding the fuel break project that enclosed ignition points for fires that spread to at least one of the fuel breaks in the project (Table 2). We adopted the term “fireshed” for the latter metric, consistent with prior use to link fire ignition locations with the resulting fire perimeter and associated impacts [52]. The intersection length was calculated by summing the length of fuel break segments that intersected a project divided by the total burned area of the same fires (i.e., the fires that encountered the project). In this way, potential differences in burn probability (Fig S2.1 in S2 Appendix) and project size among projects are removed from the metric. Each project’s fireshed area was computed by mapping the spatial extent of the fire ignitions that generated fires that intercepted the fuel break (Fig 6). From these ignition points we created an ignition density surface and eliminated the area representing the lowest 10% in ignition density in order to exclude outliers, i.e., extreme fire events. Finally, we smoothed the boundary of the resulting area to create the fireshed, resulting in 88% of ignitions falling within the fireshed, as averaged across all firesheds.

**Table 2:** Metrics used in the scenario modeling either as response metrics or objectives. All metrics summarized at the project scale.

Metric	Definition	Units	Scenarios analyzed^3^
Net revenue^1^	Value of logs at mill after subtracting harvesting, fuel treatment, and transportation costs^2^	US Dollars	All
Intersection length	The overlap between simulated wildfires and the length of the fuel break	Meters	Scenarios 1&3
Unique fire encounters	Percent of all simulated fires that intercept a fuel break at least once		Scenarios 1&3
Project geometry	Sinuosity	Index	All
Fireshed area	Area that encloses 88% of ignitions that encounter a fuel break project	Hectares	

^1^ Methods to estimate revenue have been reported in several prior publications [26,53,55] and are summarized in S1 Appendix. See Methods text for additional details.

^2^ Source: Forest Vegetation Simulator.

^3^ See Table 1.

Net revenue (Table 2) was used as both an objective in the optimization of mixed objective scenarios, and as a response variable in the pure geometry scenarios. Revenue was calculated as a residual value based on the difference between log values and the harvesting, hauling and fuel reduction costs, as described in prior studies [26,53,55]. Parameters for costs were obtained from local timber sale planning staff [Appendix C in 53] and updated in 2020 with input from the UNF (S1 Appendix). These included stratified costs for logging, transportation, and harvesting system. Pile burn treatments were assigned a cost of $1,110 per ha. The size and species composition of logs from harvesting were tracked in FVS and revenue and merchantable volume were calculated using the economics extension in FVS [56]. Average log value by species and small end diameter were obtained from dimensional timber mills within the study area. Net revenue was estimated as the revenue less costs and assigned to each FBN segment based on area weighted values to account for the different stand polygon areas in each segment (Figs S2.2 and S2.3 in S2 Appendix). As noted in prior work the estimation of net revenue omitted: 1) planning and contracting costs, 2) cost of road maintenance and construction, and 3) removal of non-merchantable volume generated from thinned stands and marginally merchantable pulpwood material. These additional components were not included because most are budgeted outside the project planning and prioritization process. We also did not consider the cost of maintaining fuel breaks over time, which will be substantial given that re-treatments will be required every 10–20 years depending on the vegetation type. The financial calculations provided approximate values adequate to examine the economic impacts of alternative project geometries and fire management tradeoffs on the UNF. See S1 Appendix for more detailed information regarding the computation of costs and revenue.

### Analysis of scenario outputs

To examine the performance of the ForSys planning model algorithm when building alternative project geometries from the FBN segments, we plotted the sinuosity index (Fig 5) against the sequential project number generated by the model. We expected that the desired geometry, radial versus linear, would be more difficult to obtain as implemented projects increase, given fewer options to aggregate segments into projects that meet the minimum area constraint, especially given the irregular shape of the UNF. We then analyzed the relationship between project geometry and the four selected response variables described above using scatter plots and correlation analyses. After recognizing limitations in the ForSys algorithm to create a perfectly ordered sequence of project geometries (identified in the model evaluation above), we re-sorted the population of projects for each scenario according to the sinuosity index to obtain an ideal geometry and used the re-ordered sequence to examine the rate at which the project firesheds expanded across the UNF for the alternative geometry scenarios. In this process the cumulative fireshed area was calculated as projects were implemented and plotted against the length of the fuel break. Finally, to examine tradeoffs between project geometry and the economics of implementation, we plotted cumulative revenue as projects were implemented for the two geometry and two mixed-objective scenarios.

## Results

### Treatments required to meet FBN fuel loads

Modeling the forest and fuel management treatment prescriptions in FVS on the portion of the FBN available for management yielded estimates of area requiring treatments to meet fuel break thresholds on the UNF (Fig 2B). We found that about 97% of the available FBN within the UNF will require thinning combined with pile burn treatments, whereas 24% requires pile and burn only (Fig 2B). About 1% of the forested area within the fuel break network met current fuels standards to qualify as a fuel break segment. About 2% was in scablands and grass shrub areas that do not support forest vegetation. Thus, UNF-wide implementation of the FBN to meet fuel loading objectives that potentially provide for ingress and egress for fire operations will require treatments on an estimated 52,896 ha.

### Optimizing alternative project geometries

**Fig 7:**
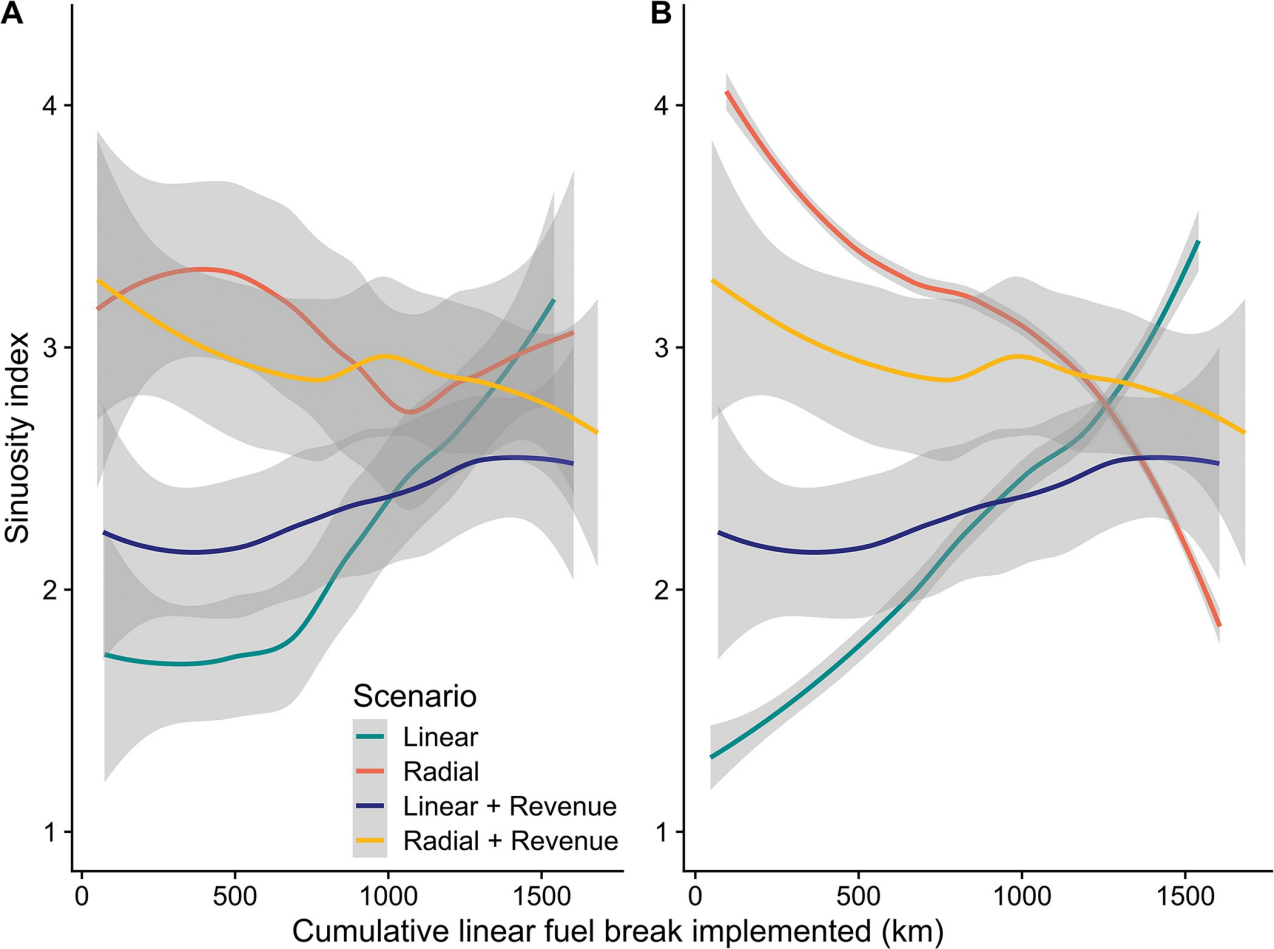
Scenario sinuosity results. A) Model outputs showing fuel break project sinuosity as successive projects are implemented for alternative geometries, and combined geometry and revenue directly from the ForSys model. Outputs are smoothed with a loess function with a span = 0.75 [57]. Unsmoothed figure version in Fig S2.4 in S2 Appendix B. B) Same as A after sorting the population of geometry-only projects (Scenarios 1 and 3, Table 1) based on the sinuosity variable to simulate an ordinal implementation based on geometry objectives. The latter sequence was adopted to simulate project implementation scenarios and analyze responses. Note that mixed-objective projects were not sorted based on sinuosity.

The FBN segments identified as requiring treatment were allocated into discrete project areas optimized for different project geometries using the ForSys model. Each project treated between 800 and 1,200 ha following operational goals set by the UNF. The model was effective at achieving desired scenarios, linear versus radial, as measured by the sinuosity index (Figs 4 and 5). However, this effectiveness declined as additional projects were added to the scenarios (Fig 7, Table 3) and the project scale sinuosity for the different scenarios showed clear differences until about 50% of the area was treated, at which point the program was unable to build the targeted geometry (Fig 7A). The degree of sinuosity in Fig 7 can be translated to project geometries using two examples shown in Fig 4 that show simulated projects with a relatively low (1.5, linear) and high (4.4, radial) sinuosity index. In terms of total treated area, the geometry scenarios (linear, radial) were able to build 32–33 projects that met the 800–1,200 ha treatment target, equivalent to treating 71–75% of the fuel break network that was both available and in need of treatment on the UNF depending on the scenario (Table 3). Stray segments remained outside project areas where the algorithm was unable to connect a sufficient number of segments to meet the project area constraint.

**Table 3:** Fuel break scenarios, area treated, total revenue, and timber volume as a result of project implementation.

Scenario	Geometry	Total area treated (ha)	Percent treated of the available land needing treatment	Total revenue (millions $)	Number of viable projects^1^	Total merchantable volume (million m^3^)
1	Radial	39,491	74.7	63.4	33	2.07
2	Radial + Revenue	40,008	75.6	74.5	34	2.25
3	Linear	37,338	70.6	65.5	32	2.01
4	Linear + Revenue	38,697	73.2	64.6	33	2.05

^1^ Viable project areas were defined as a minimum of 800 ha and a maximum of 1,200 ha of the 52,896 ha of forested lands available and needing treatment.

Addition of objectives for revenue (Fig 7) moderately degraded the resulting geometry (e.g., Linear + Revenue). Overall, the scenarios treated between 73–76% of the available area in need of treatment within the network in 33–34 projects. These results indicated that the optimization algorithm in the ForSys model was capable of building projects with a range of geometries but was less effective at perfect sequencing of the projects according to sinuosity and organizing all available treatment segments into projects. Despite these inadequacies, the model was able to generate a wide range of project geometries within the target treatment areas to examine the effect on response variables and examine economic tradeoffs.

### Effect of project geometry on response variables

**Fig 8:**
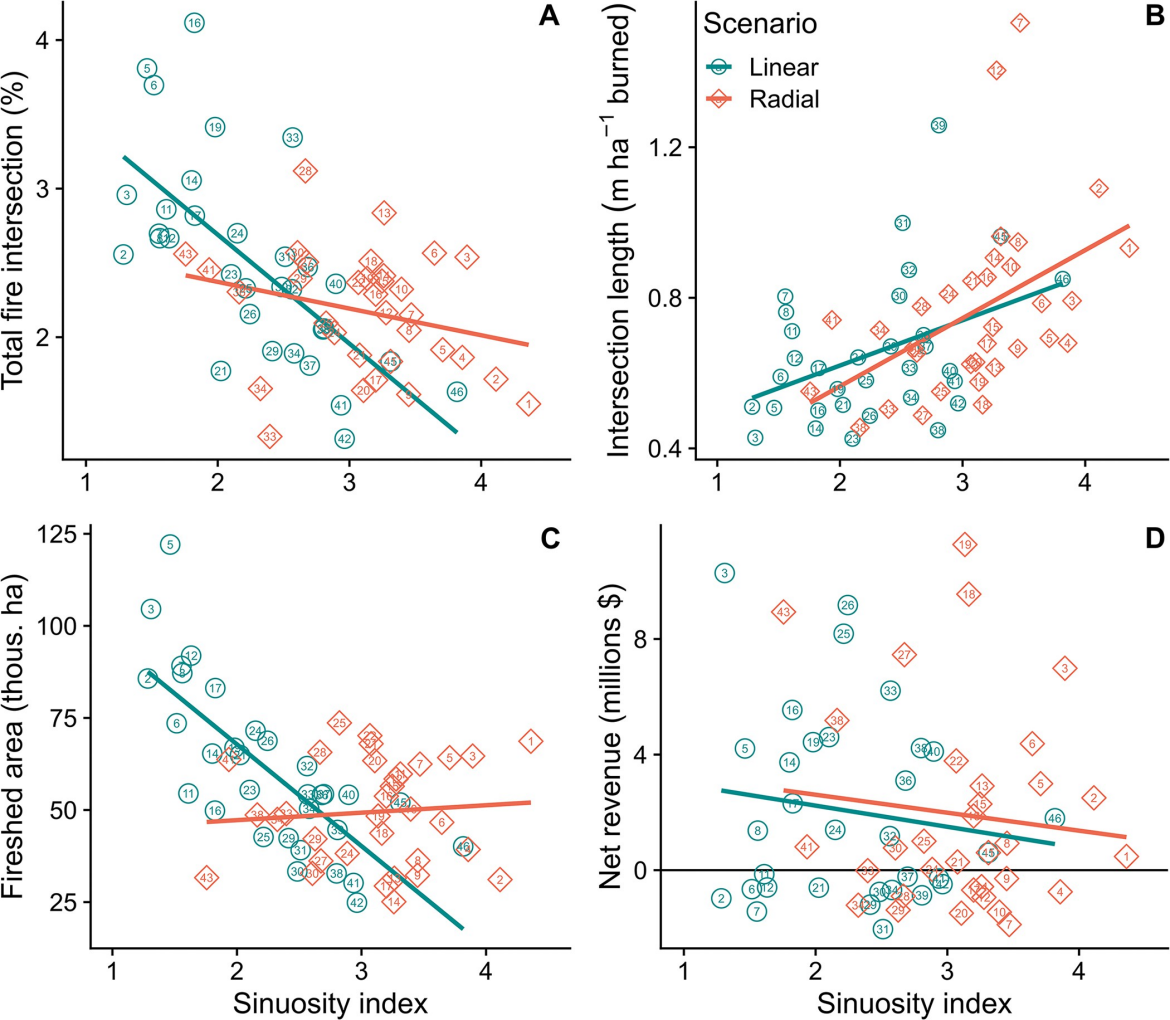
Scatter plots of project sinuosity versus four response variables for the population of projects generated by the ForSys model. A) percent of total fires that intersected the project; B) total intersection length (meters) per hectare burned for fires that intersected the fuel break segments in each project. A low value indicates few opportunities to engage in fire suppression activities, while a large value indicates higher redundancy as the same fire perimeter intersects the same project at multiple points; C) fireshed area surrounding the fuel break segments, defined as the area that encloses 88% of the ignition points for fires that intersected the project; and D) projected net revenue from forest and fuel management per project. Collectively the graphs show that radial projects have more redundancy in terms of fire overlap per unit area burned in a project, but linear projects intercept more unique fire events over a larger area of ignitions. Sinuosity index illustrated in Fig 5.

At the scale of projects, scatterplots and correlation analyses revealed that projects implemented with a more linear shape (lower sinuosity) intersected a higher number of unique fires (Fig 8A), intersected a shorter length of the FBN per ha burned in the project (Fig 8B), and were associated with larger firesheds (Fig 8C) compared to projects with a radial shape (i.e., high sinuosity). Linear projects had a lower redundancy as measured by the total intersect length (Fig 8B) in meters per ha burned ranging from about 0.4 to 1.3. Linear projects had a larger fireshed area, meaning they intercepted ignitions from a larger geographic area compared to radial projects (Fig 8C). The three projects with the lowest sinuosity intercepted fire ignitions from between 85,000 ha to nearly 125,000 ha of the surrounding area. At higher levels of sinuosity this value was reduced to approximately 50,000 ha with substantial variability among projects (Fig 8C). There was little detectible effect of geometry on potential revenue at the scale of individual projects (Fig 8D).

### Scenario implementation

**Fig 9:**
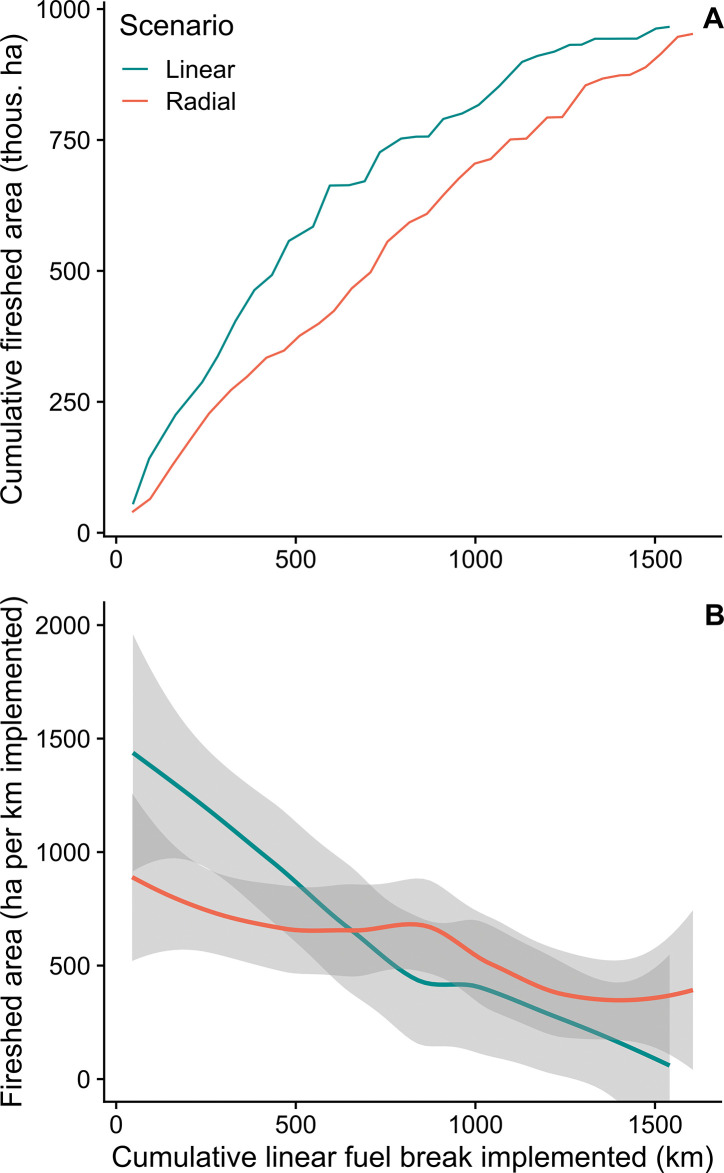
Fuel break fireshed effects. A) Cumulative area within a fuel break fireshed with increasing length of fuel breaks implemented for alternative project geometries, linear versus radial. The graph shows that for a given distance treated, linear fuel break projects have firesheds that cover more area, meaning that more fires are intercepted. The graph also shows declining addition of fireshed area when about half of the FBN is treated (1,000 km). B) Efficiency of fuel break length on fireshed area for the two geometry scenarios showing the incremental increase in fireshed area as projects are implemented. Outputs are smoothed with a loess function with a span = 0.75 [57]. See Fig 6 for definition of firesheds.

Implementation scenarios optimized for alternative geometries were created by first re-sorting the model outputs based on project sinuosity and then sequencing the projects and associated treatments. We then measured the resulting incremental expansion of the total FBN fireshed area. As explained in the Methods section, post-processing corrected for the deficiencies in the algorithm to create perfect ordinal scenarios for the respective geometries. For each scenario, the incremental, non-overlapping fireshed area was identified and used to build cumulative fireshed area graphs. The results showed that linear projects were more efficient at expanding the fuel break firesheds compared to radial ones (Fig 9A). When implementing 50% of the linear FBN (about 800 km) the fireshed area was 756,355 ha versus 592,561 ha for the radial FBN. The fireshed area increase per FBN kilometer implemented (Fig 9B) showed higher efficiency of the linear projects until about 500 km (31%) of the FBN was implemented. After this point, the firesheds of additional implemented projects increasingly overlapped firesheds of the previously implemented projects, thus resulting in a slower increase of the FBN fireshed area.

### Economic tradeoffs and efficiencies

**Fig 10:**
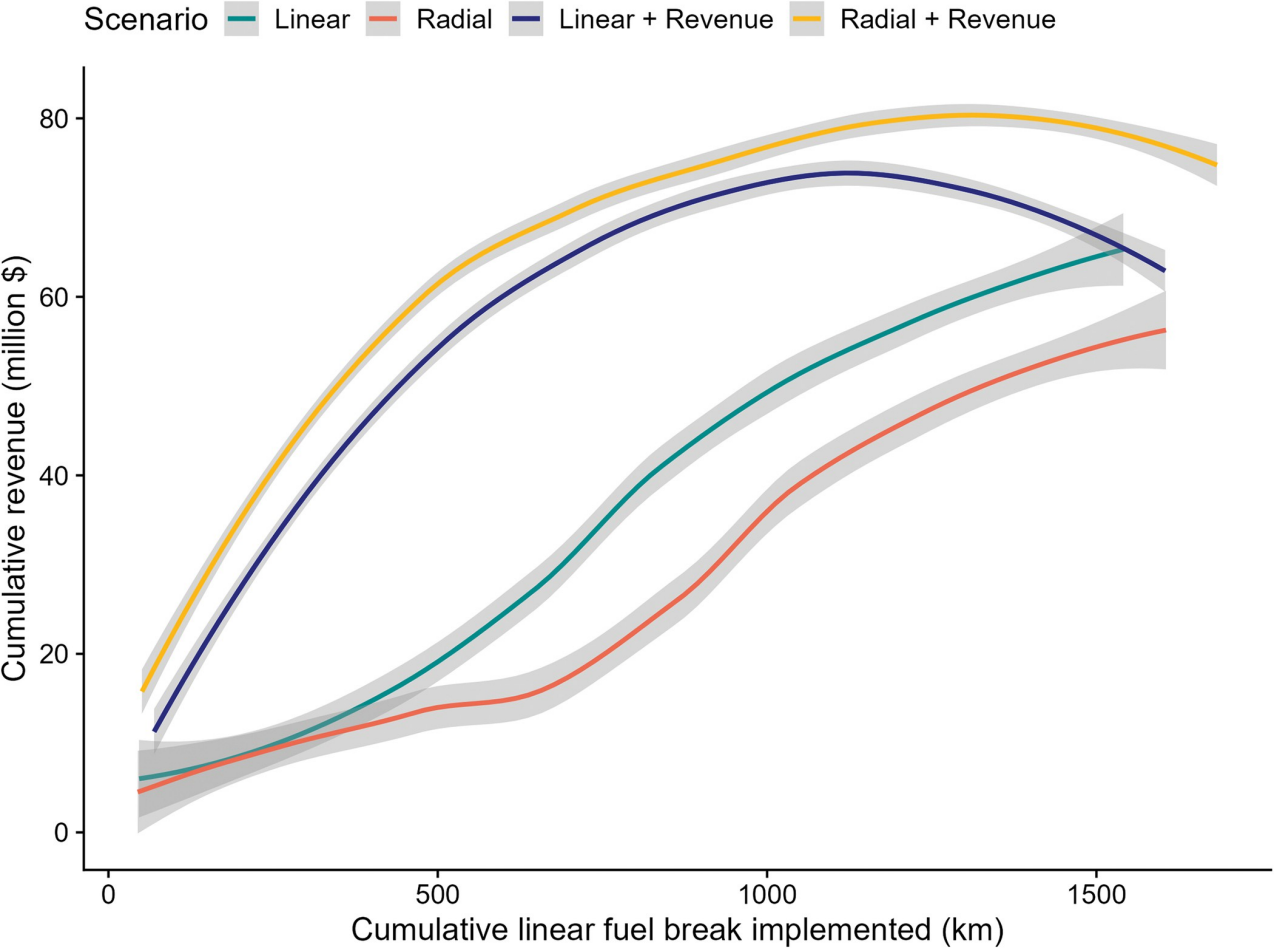
Cumulative net revenue generated from implementing a sequence of projects optimized for alternative geometries (linear versus radial) and for a combined geometry and revenue objective. Note geometry scenarios were resorted from the model outputs to create an idealized scenario sorted by sinuosity, and mixed revenue-geometry. Scenarios are graphed in the order generated from the model. Outputs are smoothed with a loess function with a span = 0.75 [57].

**Fig 11:**
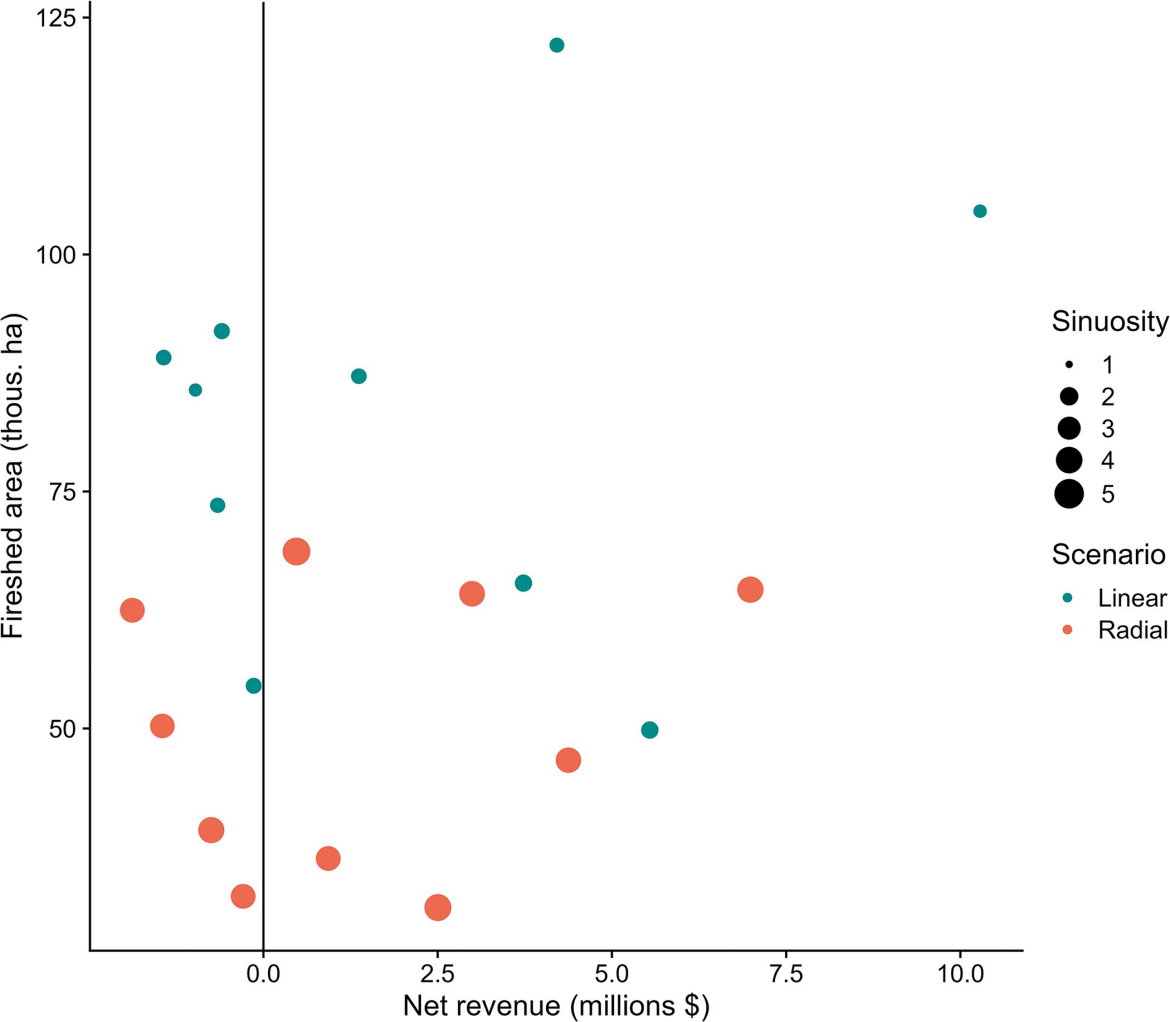
Scatter plot of fireshed area against projected net revenue from forest management per project for the linear and radial scenarios. Data are shown for 10 projects for each scenario that best achieved the desired geometry, linear versus radial, based on sinuosity. Sinuosity index illustrated in Fig 5. Fireshed area is estimated independently for each project rather than cumulatively as shown in Fig 9. See Fig 6 for definition of fuel break firesheds.

The effect of adding revenue as a weighted objective substantially increased the revenue response, generating over twice as much revenue when the first 800 km were implemented (50% of the network, Fig 10). Total revenue generated by the projects selected for the different scenarios, it ranged from a high $74.5 million for the Radial + Revenue scenario to a low of $63.4 million for the Radial scenario. Note that these revenue differences were due to the revenue composition of the subset of segments selected as part of each scenario, which ranged from 70.6% to 75.6% of the total segments. There was little detectable relationship between geometry and revenue at the scale of individual projects (Figs 8D and 11). However, the most linear projects in general had higher fireshed area, and in some cases relatively high potential revenue as well, thus providing the highest economic efficiency in terms of fireshed area and potential revenue (Fig 11).

## Discussion

We examined alternative spatial scenarios to prioritize and schedule the construction of a large-scale fuel break network similar to those being proposed in many fire impacted regions globally. The premise for the work is that correct prioritization in the early phases of implementation will have manifold effects and lasting outcomes as the remaining segments are treated, as discussed for other vegetation management systems [58]. We found that projects implemented with a more linear shape (lower sinuosity) intersected a higher number of unique fires, intersected a shorter length of the FBN per ha burned by fires encountering the project, and were associated with larger firesheds. Geometry had no consistent effect on net revenue, and including the latter as a project objective substantially increased the projected financial value of projects. The results illustrate how different prioritization strategies for implementing fuel break projects can affect performance and cost, and are relevant for both predefined and mapped FBNs yet to be implemented, as well as the design of future networks. We reiterate that proposed fuel break networks in Portugal [1,24,59], Ukraine [18], China [4], western US rangelands [20,60], and other countries [19], will take perhaps decades to implement on lands that have inherently wide variation in terms of cost effectiveness, social, environmental and ecological impacts. As a result, these investments warrant careful prioritization to ensure desired short- and long-term benefits from fuel treatment [61]. The importance of establishing and communicating spatial priorities in restoration, conservation, and risk mitigation planning has been discussed for many aspects including financial and ecological outcomes [32,62].

Specific tradeoffs between FBN design parameters, cost effectiveness, failure rates [24], and ecological impacts [20] will vary significantly among fire regimes and social-ecological systems, although literature comparing different systems is rare. In general, for given treated area, larger width improves FBN effectiveness but increases costs and potential ecological impacts. Studies have suggested the minimum firebreak width required is 1.5 times the length of the fire flame for surface fires and 2.5 times the stand height for crown fires [63]. Zong et al. [4] examined fuel break width versus density for boreal forests in China and found that under an assumed fire behavior, 30 m width fuel breaks can stop surface fire spread in grass and forests and low-intensity, intermittent crown fire in forests; 60 m width firebreaks can block crown fire spread in mixed deciduous coniferous forest; and 90 m width can stop crown fires in evergreen coniferous forest. In the western US Great Basin, a FBN is being implemented to protect habitat for the greater sage-grouse (*Centrocercus urophasianus*) [3] and local fire managers subscribe to the idea that “the wider the fuel break, the better”, although widening fuel breaks disturbs larger area with potential negative ecological consequences [64]. For instance, fuel breaks can act as a conduit for cheatgrass invasion into otherwise uninvaded locations [20,65]. Despite recommendations concerning width and density, it is important to note that the optimum width can only be described as an exceedance probability [24] since fire events that encounter a fuel break could have markedly different flame lengths depending on spread direction and fire weather. And, the effectiveness per unit width among different firebreak compositions (rock, water, treated vegetation) in terms of the effect on fire spread is not the same [10,66].

It is widely recognized that fuel breaks are not a panacea as they frequently fail to function as intended during wildfire events [14,17]. In the current study the fuel break prescription called for thinning to <15% canopy closure, eliminating the possibility of crown fire within the fuel break [45] and thereby reducing the probability of failure from spot fires burning over the 300 m wide treatments. Coupling FBNs with landscape scale forest restoration and resiliency management [67] can further improve the effectiveness of linear fuel breaks despite important differences in long-term objectives. Thus co-implementation will remain an important fuel management strategy on remote wildlands where operational economics drive treatment extent and project locations. Narrow, linear fuel breaks do not significantly contribute to broader landscape forest health and resiliency objective [67], but rather are a stopgap to prevent irreversible loss to ecosystem values by enhancing opportunities for firefighters. For instance, co-prioritization of multiple strategies [68] that couple the results in this study with that of our prior prioritization of projects to restore resiliency [26,69] and optimize the use of prescribed fire [55] within the study area are needed. Similar multifaceted approaches have been discussed for the Portuguese fuel management initiatives [59]. For instance, robust landscape restoration projects that treat 30% to 40% of the landscape, including treatments along roads, provide substantially more fire control possibilities than a FBN and thus the latter are not a priority in these areas. Conversely, portions of national forests where economic opportunities are scarce and fire issues are significant could be the priorities for FBNs.

We acknowledge many limitations of the study, some unique to our methods, and others universal among studies that use wildfire simulation to predict outcomes. In general, estimating the potential benefits of alternative prioritization schemes and spatially optimized treatments with some level of certainty is challenging given the difficulty of predicting the location, timing, and magnitude of future fire events relative to treated areas, and the availability of suppression resources to deploy at the critical fuel break locations. As in prior work [24,52], our approach to examine uncertain effects on future fires was to use a large library of simulated fires and coarsely examine their spatial interactions measured with multiple metrics. More in depth analyses are possible by re-simulating fires and examining the failure rate of the fuel break networks [18,24] and avoided losses [24], although this requires tenuous assumptions about fire intensity thresholds where fuel breaks fail or prevent suppression crews from engaging the fire at a specific point in time. A probabilistic approach to estimating failure rate used by Aparício et al. [24] is the only case study to our knowledge that factored uncertainty into the assessment of potential fuel break burn over.

We are also aware of many other prioritization metrics that could be useful to prioritize FBN treatments, including suppression difficulty indices, containment probability, and burn over rates [13,24,70]. These and other variables can be easily incorporated into our modeling framework although the effect of implementation geometry will persist. Another limitation specific to our approach is that fuel treatment projects are interdependent in terms of effects on wildfire and thus fuel break projects could change the priority of other projects in the vicinity when they are evaluated based on fire likelihood, and to a lesser extent severity, although this is not the case for financial objectives. Our sequential projects were widely spaced around the UNF and thus project to project interactions at the scale of the UNF will not materialize until a significant portion of the network is constructed. Future wildfires will change the relative priorities as well, as observed in the 2022 and prior wildfire seasons in the western US where the authors have noted a substantial number of planned and partially implemented projects burned over by wildfire. Uncertainty about future fire regimes will affect the reliability of predicted outcomes, as widely discussed in the conservation planning and restoration literature [71–73]. Thus, despite sophisticated modeling platforms like used elsewhere [e.g., 4], predicted outcomes are approximate solutions to real-world planning problems [73] and thus carry significant planning risk [58,72–74]. The wide use of probabilistic wildfire simulation to estimate wildfire risk and prioritize fuel management investments in the US and elsewhere should be tempered with the understanding that outputs carry significant uncertainty that is rarely quantified in planning and policy documents [but see 52].

Our modeling framework is easily applicable to other fire regimes and management systems to analyze priorities and design tradeoffs for proposed FBNs, and for other conservation and restoration planning problems as well [75,76]. Our future avenues of work will use artificial landscapes where selection of FBN segments is not constrained by real world features (existing roads, boundaries) such that the topological relationship between geometry and response metrics can be isolated and extrapolated to a wider array of fire regimes and management systems. In this way future planning efforts can draw from these underlying relationships to optimize implementation scenarios without detailed modeling efforts as demonstrated here.

## Supporting information

S1 Appendix Economics and modeling methodology. (PDF)

S2 Appendix Supplementary figures and tables. (PDF)
