# Supplementary material for: Optimizing the implementation of a forest fuel break network
Source: PLoS One. 2023 Dec 13;18(12):e0295392. doi: 10.1371/journal.pone.0295392 (PMC10718465; doi:10.1371/journal.pone.0295392)
Supplement: S1 Appendix — (PDF) [file pone.0295392.s001.pdf]

S1 Appendix. Economics and modeling methodology

Treatment costs

Harvest costs

Harvest costs were stratified by size class by diameter at breast height (DBH) and number of trees per acre (TPA), and by the slope of the fuel break segments. Harvest cost was based on size, and TPA was calculated in FVS using the LANFIN model, with costs assigned as in Tables A1 and A2. Average slope per fuel break network (FBN) segment was calculated from digital elevation data (30 x 30 m) in ArcGIS Pro, using the Zonal Statistics as Table tool. A ground-based harvesting system was assigned to stands with a slope ≤35%, and a cable-based harvesting system was assigned for all stands with slopes >35%.

Table S1.1. Ground-based logging activity cost ($/m^3) by tree size class and number of cut trees per acre used to estimate harvesting costs for each simulated project area.

	Number of cut trees per hectare					
**Tree size, DBH (cm)**	**12**	**49**	**124**	**247**	**494**	**988**
0.0 – 15					295	275
15 – 17.5				241	221	206
17.5 – 22.7			190	173	161	153
22.7 – 40.3		116	108	108	102	102
40.3 – 53.3	108	108	108	108	102	99

Ground-based logging activities were assumed in stands with an average slope <35%.
Harvest cost estimates were adopted from Rainville et al. [1].

Table S1.2. Cable-based logging activity cost ($/m^3) by tree size class and number of cut trees per acre for each simulated project area.

	Number of cut trees per hectare					
**Tree size, DBH (cm)**	**12**	**49**	**124**	**247**	**494**	**988**
0.0 – 15					1042	728
15 – 17.5				1090	657	504
17.5 – 22.7			838	586	399	337
22.7 – 40.3		244	232	212	207	204
40.3 – 53.3	201	167	161	161	158	158

Cable-based logging activities were assumed in stands with an average slope >35%.
Harvest cost estimates were adopted from Rainville et al. [1].

Hauling costs

Timber hauling costs were calculated from each individual stand to the nearest wood processing facility and were estimated using the road network classified by driving speed. Round-trip travel time between each stand and the nearest processing facility was computed for the shortest path using travel distance and road-specific speed [2] (Table A3). Round trip costs per cubic meter of timber were estimated using travel time, truck hourly cost of $100 at 10 hours per day, and truckload capacity of 12 m^3. One additional hour of delay time was added for loading, unloading and wait times. Note that hauling cost was only included in the net revenue calculation for stands that generated more than 35 m^3 ha^{-1} of merchantable timber, and reflected the round trip distance to the nearest mill.

Hauling costs were calculated as:

$$(loading\ time\ (hr) + travel\ time\ (hr)) \times \frac{\$100.00}{hr} \times \frac{Total\ merch.volume\ (m^3)}{12\ m^3\ per\ truck} \times 2 \qquad (1)$$

Table S1.3. Assumed average haul speeds by road maintenance class.

Road Class	Speed class (km hr-1)
Interstate	105
State	72
Secondary state	56
Gravel	40
Dirty	12
No Road	5

Revenue from treatments

Harvested timber volume from fuel treatments

We attributed the FBN segments with forest vegetation data by intersecting the 300 m wide FBN with the Forest vegetation polygons [3]. Inventory data for each stand was obtained from the Forest Service FSVeg inventory data system [3]. The vegetation polygons were originally delineated from photointerpretation and follow natural breaks in vegetation type and changes in stand structure from past management activities and disturbance. As noted in the methods, we did not distinguish the road surface area in the spatial configuration since roads are represented as line features in the forest GIS layers. This results in a small bias in the area calculations that does not significantly affect the results or conclusions. The resulting stand polygon layer that intersected the FBN contained 57,606 features, ranging in size from 0.00001 ha to 16.84 ha with an average of 3.0 ha. Seven stand slivers in the initial dataset had no calculable area and were excluded from the analysis. The final FBN segment polygon layer contained a total of 2766 features, ranging in size from 33.0 ha to 106.1 ha, with an average size of 71.3 ha.

Harvested volume was estimated by simulating treatments designed by the forest fuels planner (Stinchfield pers comm.) with the Forest Vegetation Simulator Blue Mountains variant (Keyser and Dixon, 2015). The thinning treatment consisted of a thin from below followed by a pile and burn and was assigned to all stands with canopy cover exceeding 15%. Thinning from below prioritized removal of smaller trees of targeted fire-intolerant species (e.g., grand fir) and reduced ladder fuels to prevent torching and crowning fire behavior. Grand fir was thinned from below up to a maximum DBH of 76.2 cm, while the maximum harvested tree size for other species was set to 53.3 cm to meet late-old structure objectives [4]. Conifer stands that did not meet the canopy cover threshold, but had a 20% or greater chance of > 1.2 m flame lengths received a pile and burn treatment consisting of hand or machine piling of harvest residue and downed woody material as practiced on the Forest, at a cost of $1110/ha. The pile and burn treatments were simulated using the FuelMove keyword, which has the same effect as the pile burn process in terms of removing fuels from the site. Note that the thinning prescription is specific to fuel breaks and outside of these areas treatments are customized to individual plant association groups to meet restoration objectives [5].

Log Values

Average pond values by species and small end diameter for all dimensional timber mills within the study area were collected from timber sales specialists on the Umatilla National Forest, and updated in 2020 (Tables A3, A4). We calculated the small end diameter of each harvested tree by bucking the stem and generating logs using the economics extension of FVS [6].

Table S1.4. Delivered log values ($/m^3) by species and size class used in the estimation of revenues from thinning treatments for the northern Umatilla National Forest.

	Species			
Small-end diameter (cm)	Douglas-fir, western larch	Hemlock spp., fir spp.	Ponderosa pine	Lodgepole pine, Spruce spp.
15.2-20.3	1175	1147	820	956
20.3-33.0	1175	1147	860	956
33.0-45.7	1175	117	928	1093
45.7 +	1093	1065	1024	1093

Table S1.5. Delivered log values ($/m^3) by species and size class used in the estimation of revenues from thinning treatments for the southern Umatilla National Forest.

	Species			
Small-end diameter (in)	Douglas-fir, western larch	Hemlock spp., fir spp.	Ponderosa pine	Lodgepole pine, Spruce spp.
6.0-7.9	615	751	615	820
8-12.9	820	820	820	820
13-17.9	820	874	820	820
18 +	888	888	888	820

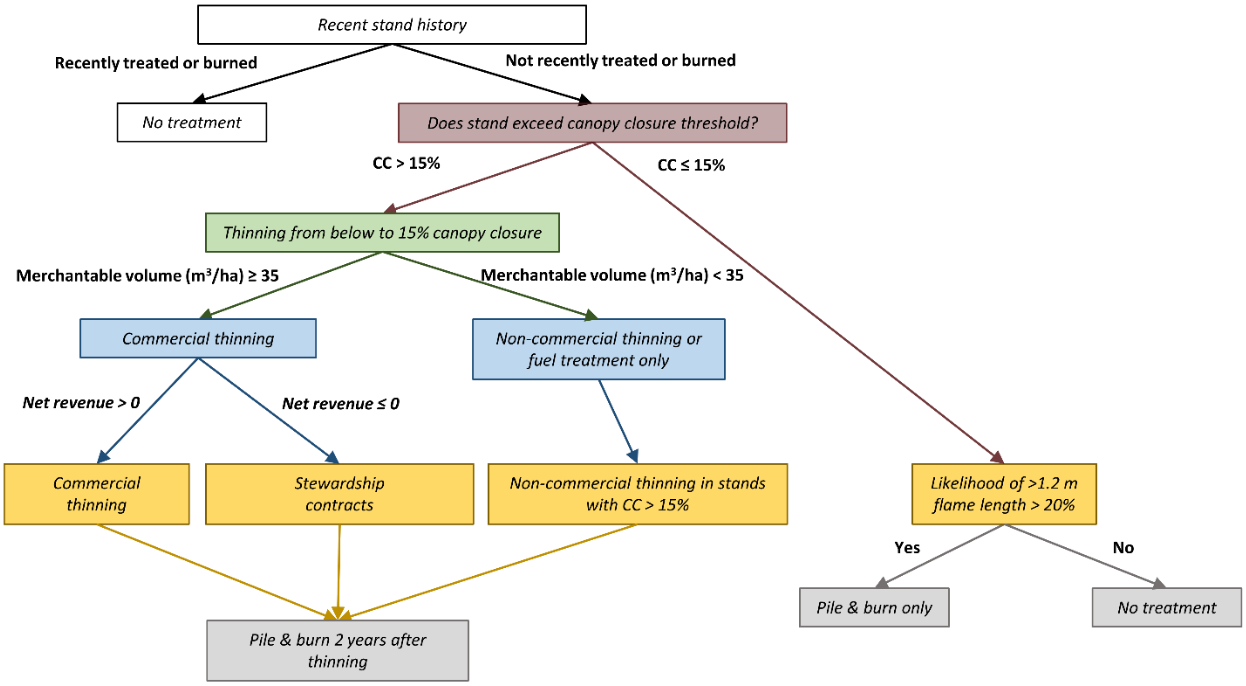

Fig S1.1. Decision-tree used to assign treatment prescription to each stand based on stand canopy closure (CC), merchantable volume, net revenue and flame length likelihood. Adapted from Belavenutti et al. [7].

Optimization model

We used the ForSys model developed from the Landscape Treatment Designer [LTD, 8, 9]. The input file consisted of a shapefile containing the outputs from treating each 300 m segment of the fuel break and a matrix of adjacency for each segment. The program then tests each segment as a seed for a project (1200 ha of treatments) and adds adjacent segments. Intersections provide a choice for the algorithm. The process is repeated for each segment and the project that maximizes the objective value is reported.

$$\text{Max} \sum_{j=1}^{k}\left(Z_j * \sum\left(W_i N_{ij}\right)\right) \tag{2}$$

Subject to:

$$\sum_{j=1}^{k}\left(Z_j A_j\right) \leq C \tag{3}$$

where C is a global constraint of 1200 ha, Z is a vector of binary variables indicating whether the jth segment is treated (e.g., Z_j = 1 for treated segments and 0 for untreated segments), N_{ij} is the contribution to objective i in segment j if treated, and A is the area of the jth treated stand. W_i is a weighting coefficient that can be used to emphasize the objective versus distance from the project centroid (seed polygon) thus creating different levels of linearity. We prioritized projects and noted the point at which 1200 ha of adjacent segments were not

available for each scenario. Constraint slack was set to 400 ha so that every segment was prioritized into a project if it could sum to a minimum of 800 ha treated.

References

1. Rainville R, White R, Barbour J. Assessment of timber availability from forest restoration within the Blue Mountains of Oregon. Gen. Tech. Rep. Portland, OR: USDA Forest Service, Pacific Northwest Research Station, 2008 PNW-GTR-752.
2. Dijkstra EW. A note on two problems in connexion with graphs. Numerische mathematik. 1959;1(1): 269-71.
3. USDA Forest Service, Natural Resource Manager. FSVeg Common Stand Exam User Guide. 2.12.6 ed: USDA Forest Service; 2015.
4. USDA, USDI. Record of decision for amendments to Forest Service and Bureau of Land Management Planning departments within the range of the Northern Spotted Owl. Portland, OR: USDA Forest Service and USDI Bureau of Land Management, 1994 April 13, 1994. Report No.
5. Ager AA, Day MA, Vogler K. Production possibility frontiers and socioecological tradeoffs for restoration of fire adapted forests. Journal of Environmental Management. 2016;176: 157-68. doi: 10.1016/j.jenvman.2016.01.033.
6. Martin F. User Guide to the Economic Extension (ECON) of the Forest Vegetation Simulator. Fort Collins, CO: U. S. Department of Agriculture, Forest Service, Forest Management Service Center, 2013.
7. Belavenutti P, Chung W, Ager AA. The economic reality of the forest and fuel management deficit on a fire prone western US national forest. Journal of Environmental Management. 2021;293: 112825. doi: 10.1016/j.jenvman.2021.112825.
8. Ager AA, Vaillant N, Owens DE, Brittain S, Hamann J. Overview and example application of the Landscape Treatment Designer. Gen. Tech. Rep. Portland, OR: USDA Forest Service, Pacific Northwest Research Station, 2012 PNW-GTR-859.
9. Ager AA, Vaillant NM, McMahan A. Restoration of fire in managed forests: a model to prioritize landscapes and analyze tradeoffs. Ecosphere. 2013; 4(2): 29. doi: 10.1890/ES13-00007.1. Available from: http://dx.doi.org/10.1890/ES13-00007.1.
