# Supplementary material for: Optimizing the implementation of a forest fuel break network
Source: PLoS One. 2023 Dec 13;18(12):e0295392. doi: 10.1371/journal.pone.0295392 (PMC10718465; doi:10.1371/journal.pone.0295392)
Supplement: S2 Appendix — (PDF) [file pone.0295392.s002.pdf]

S2 Appendix. Supplementary figures and tables

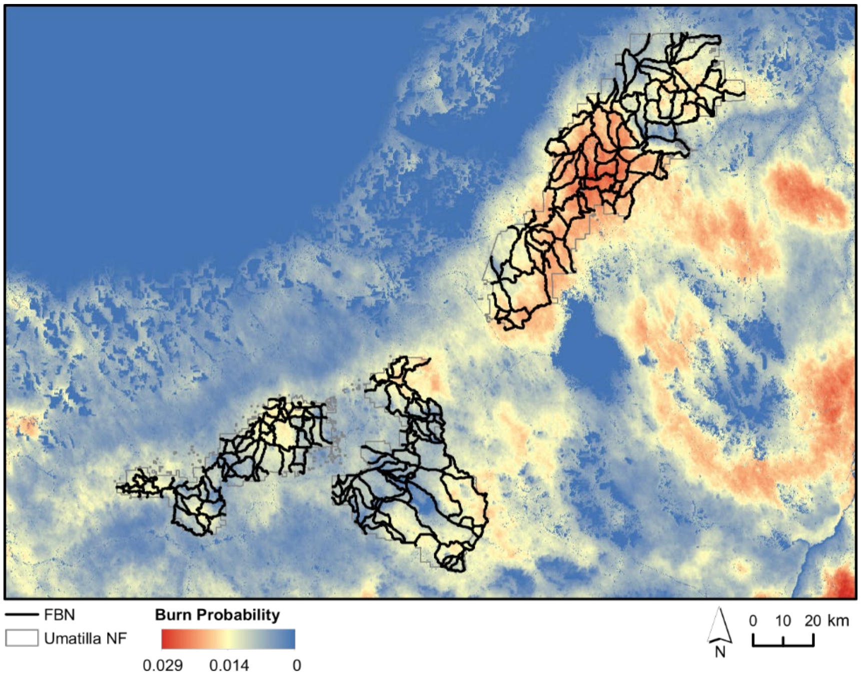

Fig S2.1. Burn probability map for the study area generated from FSim wildfire simulations and LANDFIRE fuels data. Simulations were conducted at 270 x 270 m pixel resolution using LANDFIRE data aggregated from the original 30 x 30 m pixel data. The values represent the annual probability of a pixel burning. Detailed description of the FSim simulations and 32,894 underlying fire perimeters can be found in Belavenutti et al. [1], Helmbrecht [2] and Belavenutti et al. [3].

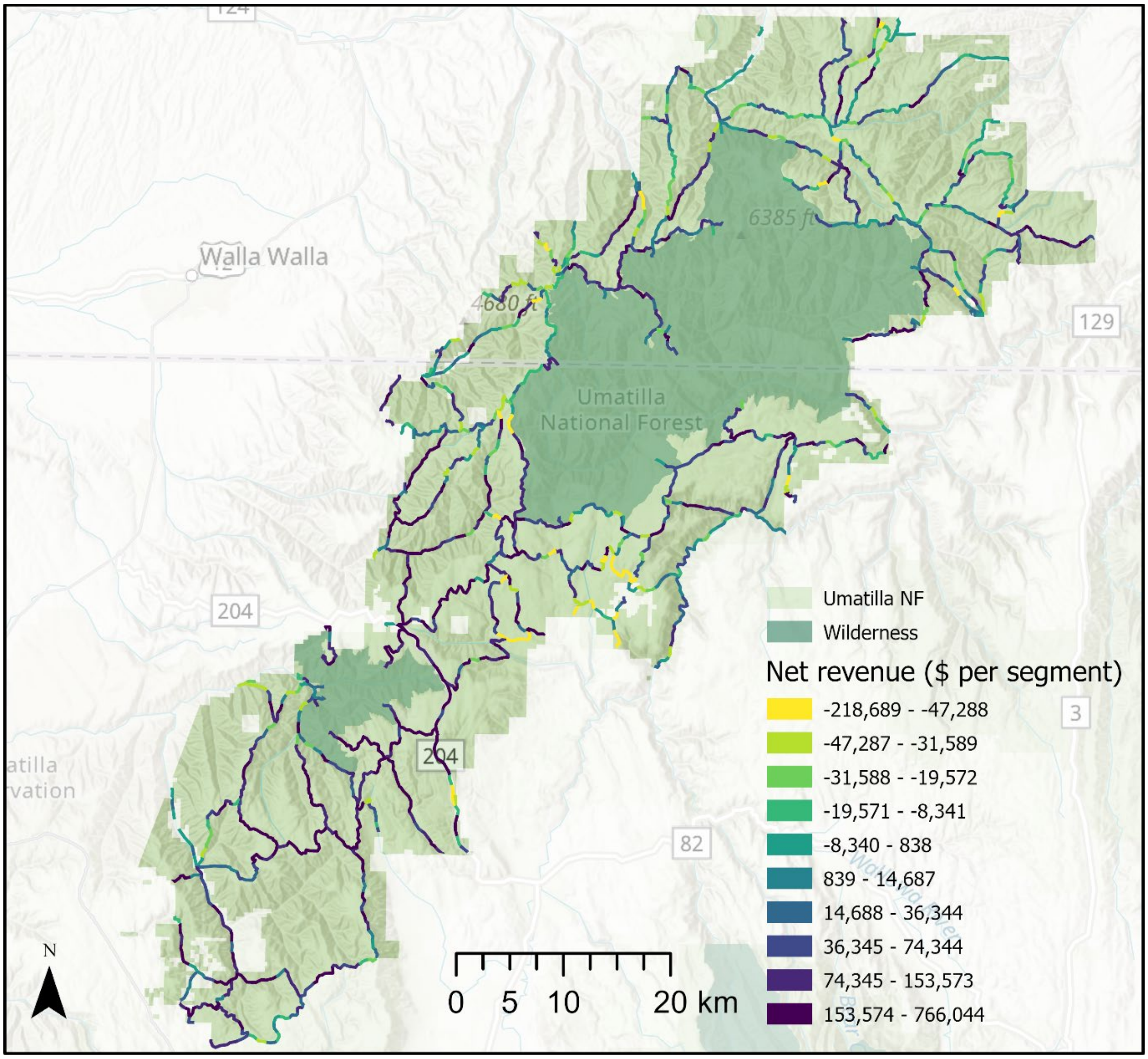

Fig S2.2. **Distribution of projected revenue from treatments for the northern portion of the forest.** See Fig S2.3 for the southwestern portion of the forest. Revenue is projected to be negative in areas where the value of the logs transported to the nearest mill is less than the sum of the harvesting, transportation, and pile and burn costs. Stands that have no commercial timber still incur a pile and burn cost also contributing to negative net revenue.

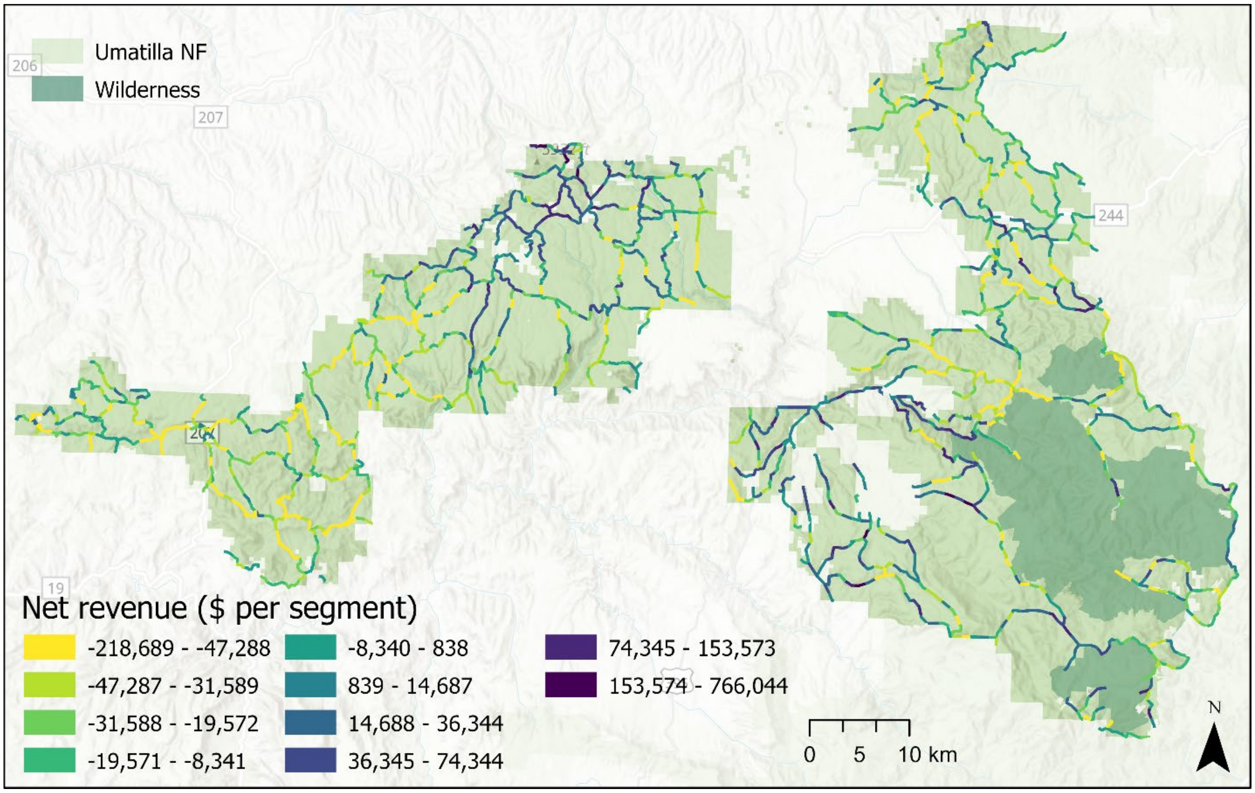

Fig S2.3. Distribution of projected revenue from treatments for the southwestern portion of the forest. See Fig S2.2 for the northern portion of the forest. Revenue is projected to be negative in areas where the value of the logs transported to the nearest mill is less than the sum of the harvesting, transportation, and pile and burn costs. Stands that have no commercial timber still incur a pile and burn cost also contributing to negative net revenue.

Table S2.1. Fuel break network area by ownership and treatment type required. Area values are presented as the percentage of the entire network and of the lands available for treatment. Data corresponds to Fig 2.

		Area		
**Ownership**	**Treatment**	**Hectares**	**Percent of total**	**Percent of available NF**
Non-NF	NA	41,713	38.0	NA
NF	Protected	11,748	10.7	NA
	Requires thinning and pile + burn	39,441	36.0	70.2
	Requires pile + burn only	13,455	12.3	23.9
	Desired condition (grass/shrub)	2471	2.3	4.4
	Desired condition (forest)	831	0.8	1.5

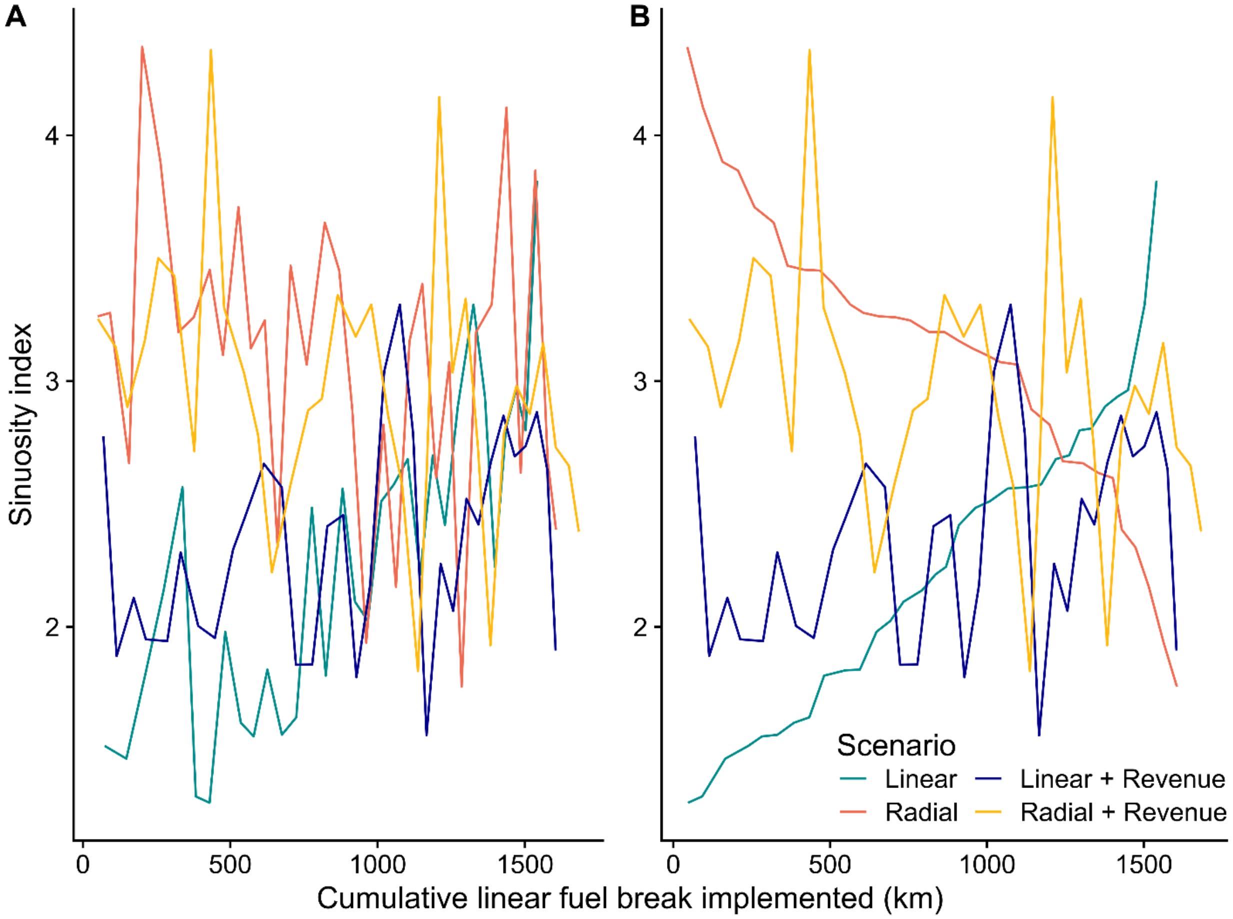

Fig S2.4. Scenario sinuosity results. A) Model outputs showing fuel break project sinuosity as successive projects are implemented for alternative geometries, and combined geometry and revenue directly from the ForSys model. Data are smoothed in Fig. 6. B) Same as A after sorting the population of geometry-only projects (Scenarios 1 and 3, Table 1) based on the sinuosity variable to simulate an ordinal implementation based on geometry objectives. The latter sequence was adopted to simulate project implementation scenarios and analyze responses. Note that mixed-objective projects were not sorted based on sinuosity.

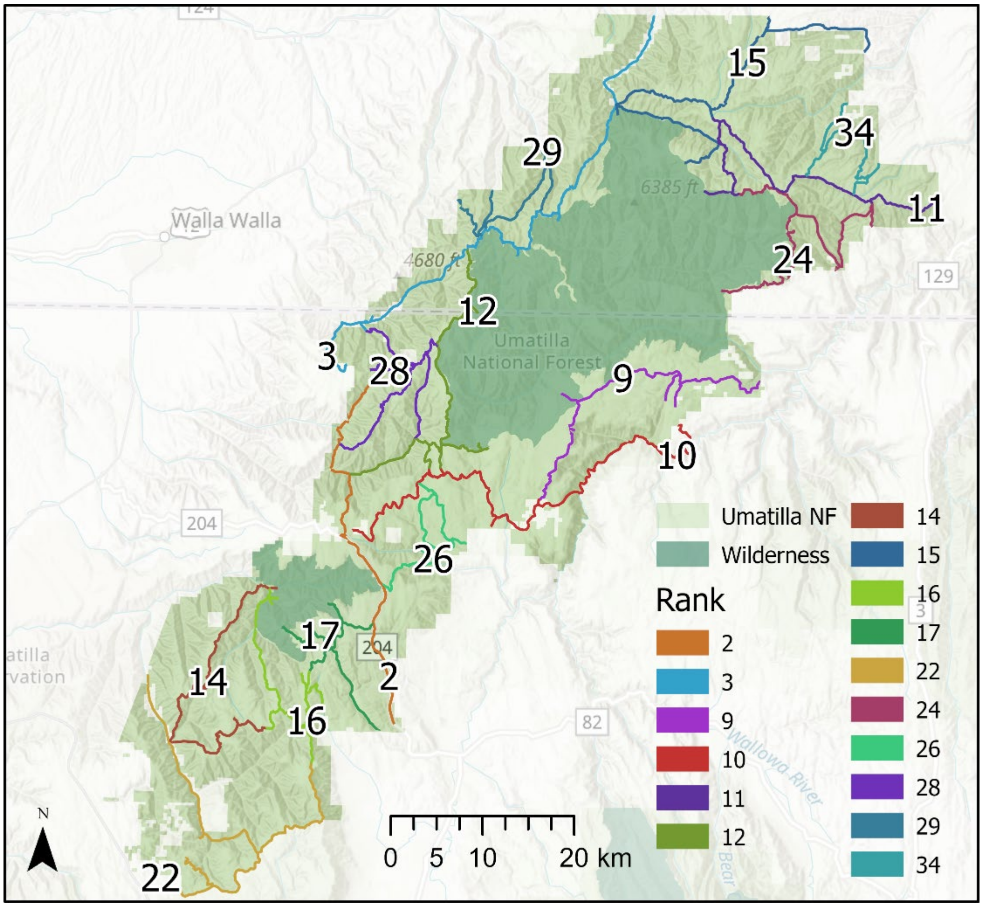

Fig S2.5. Fuel break network project rank for the Linear scenario for the northeastern portion of the study area. See Fig S2.6 for the southern portion of the forest. Projects were resorted with the highest rank (#1) assigned to the lowest sinuosity project.

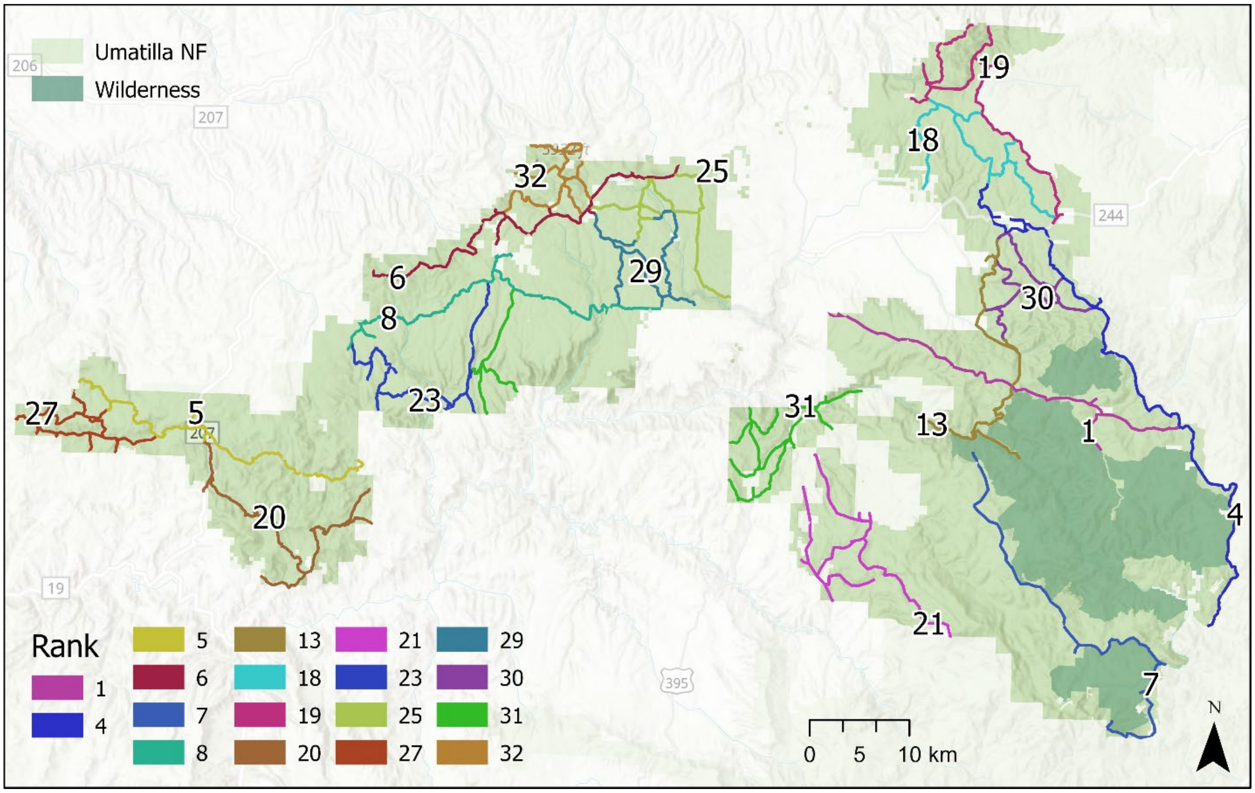

Fig S2.6. Fuel break network project rank for the Linear scenario for the southwestern portion of the study area. See Fig S2.5 for the northern portion of the forest. Projects were resorted with the highest rank (#1) assigned to the lowest sinuosity project.

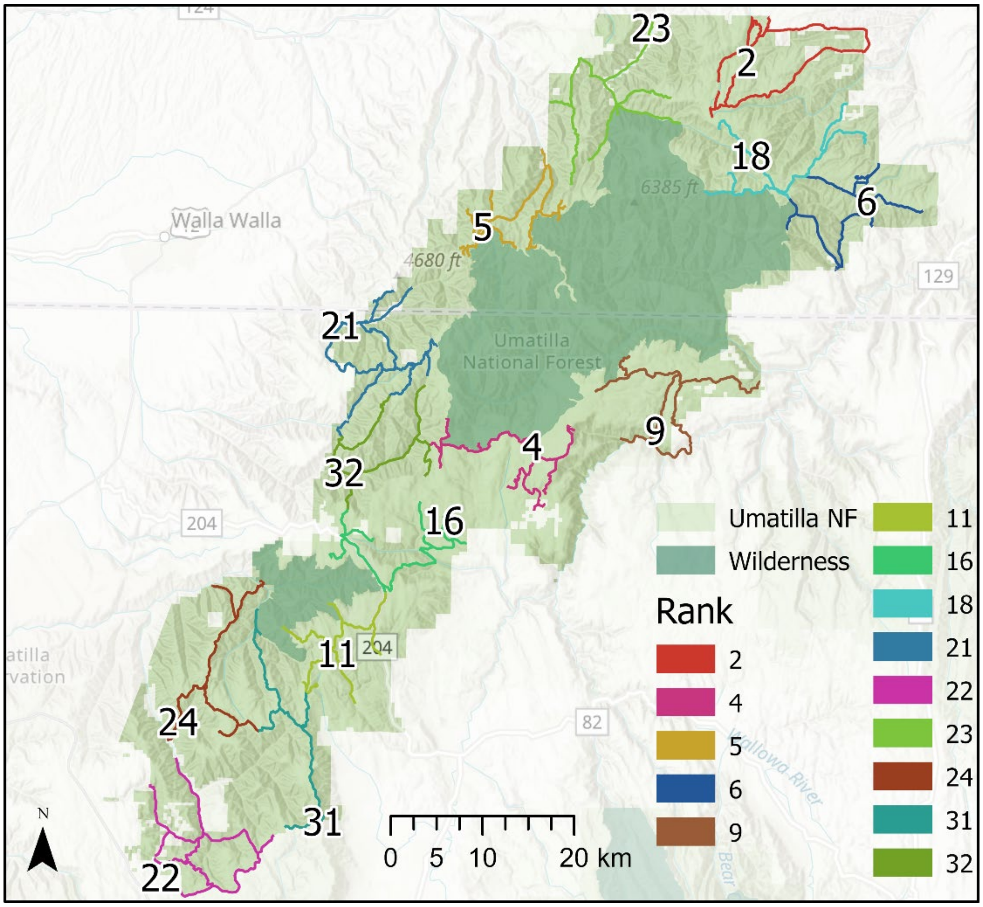

Fig S2.7. Fuel break network project rank for the Radial scenario for the northeastern portion of the study area. See Fig S2.8 for the southern portion of the forest. Projects were resorted with the highest rank (#1) assigned to the highest sinuosity project.

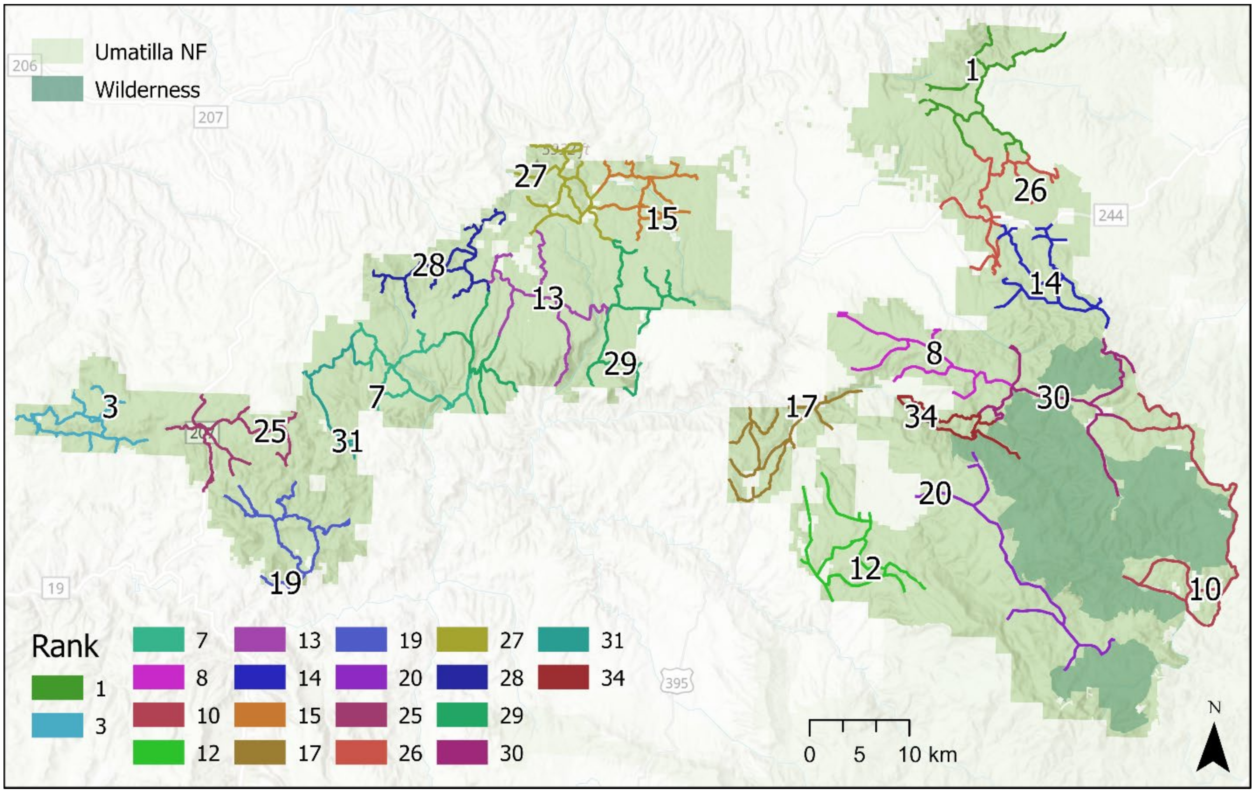

Fig S2.8. Fuel break network project rank for the Radial scenario for the southwestern portion of the study area. See Fig S2.7 for the northern portion of the forest. Projects were resorted with the highest rank (#1) assigned to the highest sinuosity project.

References

1. Belavenutti P, Chung W, Ager AA. The economic reality of the forest and fuel management deficit on a fire prone western US national forest. Journal of Environmental Management. 2021;293: 112825. doi: 10.1016/j.jenvman.2021.112825.
2. Helmbrecht D. Blue Mountains forest resiliency project: Quantitative wildfire risk assessment. Prepared for the Ochoco, Umatilla, and Wallowa-Whitman National Forests. USDA Forest Service, 2019.
3. Belavenutti P, Ager AA, Day MA, Chung W. Designing forest restoration projects to optimize the application of broadcast burning. Ecological Economics. 2022;201: 107558. doi: 10.1016/j.ecolecon.2022.107558.
